# Application of acceptability in dietary optimisation models: a systematic review

**DOI:** 10.1017/S1368980026102717

**Published:** 2026-05-22

**Authors:** Cathrine Baungaard, Magaly Aceves-Martins, Graham Horgan, David Little, Jonathan Hillier, Baukje de Roos

**Affiliations:** 1 Rowett Institute of Nutrition & Health, University of Aberdeen, UK; 2 Biomathematics and Statistics Scotland, UK; 3 Institute of Aquaculture, University of Stirling, UK; 4 Global Academy of Agriculture and Food Systems, University of Edinburgh, UK

**Keywords:** Acceptability, Sustainable diet, Mathematical optimisation, Diet optimisation, Food choice, Culturally relevant diet

## Abstract

**Objective:**

Diet optimisation models have been developed to deal with the complexities of designing a sustainable diet. Diet acceptability is one aspect of a sustainable diet that has had limited integration into these models. This systematic review summarises and critically assesses the available literature regarding diet acceptability in optimisation studies.

**Design:**

A systematic search on Medline and EMBASE was implemented, followed by a snowballing search until no new articles were found. Inclusion criteria were as follows: (1) diet optimisation studies; (2) acceptability included in model; (3) outcome diet referred to as ‘acceptable’; (4) published between 2013–2025.

**Setting:**

Diets.

**Participants:**

Adult population.

**Results:**

A total of forty-nine studies were included in this review. Definitions of acceptability varied considerably. Analysis of the studies found six overarching themes were used to define acceptability in diets, with minimisation of deviation from observed diet and setting of lower and upper bounds on intake being most commonly used. Only 25 % of studies assessed the impact of acceptability models, objective functions and/or constraints, on the dietary output to some degree. Assessing three pathways (deduction, translation and induction) for defining and operationalising acceptability presents new opportunities to include citizens in the development of acceptable dietary models.

**Conclusions:**

Including acceptability into diet optimisation models is imperative as they can influence dietary structure, nutritional content, price and environmental impact, although they present certain trade-offs compared to other optimisation goals. Further work on real-world testing and contextualisation of these often-theoretical diets is needed, alongside testing and justification of the models’ parameters for acceptability.

Rapid and immediate global transformation of food systems is key to simultaneously achieving the Sustainable Development Goals^(1)^, the transition to ‘net zero’^(2)^ and addressing the biodiversity^(3)^ and global health crises^(4)^. However, a transition of food systems will not be achieved without demand-side measures, such as shifting to healthy and sustainable consumption patterns (sustainable diets)^(5,6)^. Officially defined by the FAO of the United Nations in 2010^(7)^ and updated in 2019, sustainable diets have been described as dietary patterns that promote human health and wellbeing, reduce environmental pressures, are accessible, affordable, safe, equitable and culturally acceptable^(8)^.

Many approaches have been developed to study the composition of improved diets, one of which is mathematical optimisation modelling^(9)^. This approach has recently been proposed as the method of choice for creating diets that consider the trade-offs between health, environment and cost^(9,10)^ and offers a more efficient alternative to traditional substitution or iterative methods based on expert judgement, which lack a guaranteed optimal solution^(11)^. Initially, diet optimisation studies were modelled solely for nutrition^(12)^; however, over the years economic and environmental constraints have been included^(10,13)^, evolving to better reflect the definition of a sustainable diet^(8)^. Diet optimisation studies have shown it is mathematically possible to achieve diets that are nutritious, economically affordable, ‘acceptable’ and lower in greenhouse gas emissions^(14–16)^. While there are a myriad of ways of achieving sustainable diets^(17)^, most optimised diets would require significant shifts from existing diets^(18–20)^ or include foods not yet widely available or familiar^(15)^, which limits their acceptability^(21–23)^. This is especially pertinent for specific food groups that are key to transitioning towards more environmentally friendly diets, for example legumes and pulses^(24)^ and certain types of aquatic foods^(25)^. Both food groups have important cross-cultural distinctions which can be disaggregated across regions, traditions and consumer groups^(26,27)^.

Optimisation modelling studies are often foundational to dietary recommendations, and as such, it is imperative that the modelled diets are acceptable^(28)^. The call for cultural adaptations of environmentally friendly diets has also come from the authors of the EAT-Lancet planetary health diet^(24)^ and others^(29)^. It is important that these frameworks are understood so that the solutions from optimisation studies can be successfully integrated into food system metrics^(30)^, interventions and policy recommendations. Without acceptability, there is a risk that diets modelled for sustainability are rejected and do not receive the uptake that is required for food systems transformation. There is an increasing recognition that sustainable diets should be adaptable, tailored to cultural preferences and the contextual differences of populations^(17,29,31)^, and that modelled diets are grounded in this practical adoption^(13,28,32,33)^, especially as studies have shown that without acceptability constraints optimised diets are not feasible to implement^(31,34)^.

Acceptability in diet optimisation is not a new phenomenon^(35)^. In 1959, Smith^(36)^ first highlighted the need to consider tastes, palatability and habits, but also emphasised the difficulty of such an ‘*unquantifiable*’ and ‘*subjective’* variable. Smith included minimum amounts of foods to make sure they were included in the models^(36)^. Almost eight decades later, the assessment of the ‘practical adoption’, ‘realistic nature’ and ‘acceptable’ changes required from optimised diets at the population, sub-population and individual level still requires a much better understanding^(13)^. Previous reviews have briefly discussed acceptability in the wider context of linear programming (LP) for diet optimisation^(9,10,13,17,37)^. However, a detailed review of available literature on the concept of acceptability in dietary modelling is absent. As such, with this review we echo the call of House *et al.* ^(28)^ for more attention for the connection between acceptability and sustainability and its role in food system. By focusing on the more methodological aspects, we aim to understand the definition of the concept of acceptability in diet optimisation models, with the purpose of providing recommendations for incorporating acceptability into dietary modelling and specifically sustainable dietary modelling.

## Methods

This review used the Preferred Reporting Items for Systematic Reviews and Meta-Analyses guidelines^(38)^ as a standard for conducting a systematic search on digital databases. The Participant, Intervention, Comparator, Outcome framework was used to define how acceptability is defined and practically applied in the dietary optimisation literature (Table 1).

**Table 1.** PICOS inclusion and exclusion criteria Table 1 long description.

PICOS criteria	Inclusion	Exclusion
Population	Adults (+18 years).	People (< 18 years), medical population, families with individuals < 18 years.
Intervention	Dietary modelling studies that included acceptability as an objective function, constraint or claimed modelled diet was acceptable. Published in the last 10 years from May 2013.	No mention of acceptability, or published before 2013.
Comparator	NA	NA
Outcome	Methods of designing and definition acceptability and how this impacts dietary solutions.	No mention of acceptability in model or in contextualising the model.
Study design	Dietary optimisation studies. Publications in English language only.	Non-diet optimisation studies, optimisation for animals and non-English publications.

### Methods for evaluating acceptability

Food choice and food or diet acceptability decisions are complex behaviours, influenced by many factors, with personal values and attitudes playing a strong role^(39,40)^. As such, this review took a structuralist theoretical perspective. This stance makes the assumption that social institutions (e.g. societies, cultures, systems and networks) and physical structures (e.g. markets, governments and places) influence food choice and acceptability by either enhancing or limiting an individual’s food decision, through the norms and values embedded within these structures^(41)^. With this interpretative framework in mind, we used the following definitions.

*Food choice* refers to how an individual decides what foods to eat. These decisions are taken frequently, yet are complex and influenced by many factors and change over time^(41)^. *Food acceptability* is often referred to by various terms such as liking/disliking, food preferences, palatability and generally relies on either psychometric, psychophysical and/or behavioural methods to measure^(42)^. Conversely, Caspi *et al.* ^(40)^ defined *food acceptability* as the attitudes an individual has regarding the characteristics of the local food environment, as well as whether the products supplied meet their individual standards. These personal standards, attitudes or preferences are influenced by social and physical structures and provide individuals with reference points to assess and judge whether their behaviours are ‘acceptable’ or ‘unacceptable’^(41)^. These standards will vary among populations, subgroups and individuals and over the course of time^(41)^. This review considers *food choice* as an aspect of *acceptability,* but not a proxy. While the cost of a diet or food item is a part of the complex interactions of determinants for diet acceptability^(43,44)^, diet cost was not assumed as part of the definition of ‘acceptability’, since dietary cost is a common constraint already generally integrated into dietary optimisation models^(13,37)^. This review considers dietary cost as a constraint separate from acceptability and is therefore outside the scope of this review.

### Literature search

**Figure 1.**
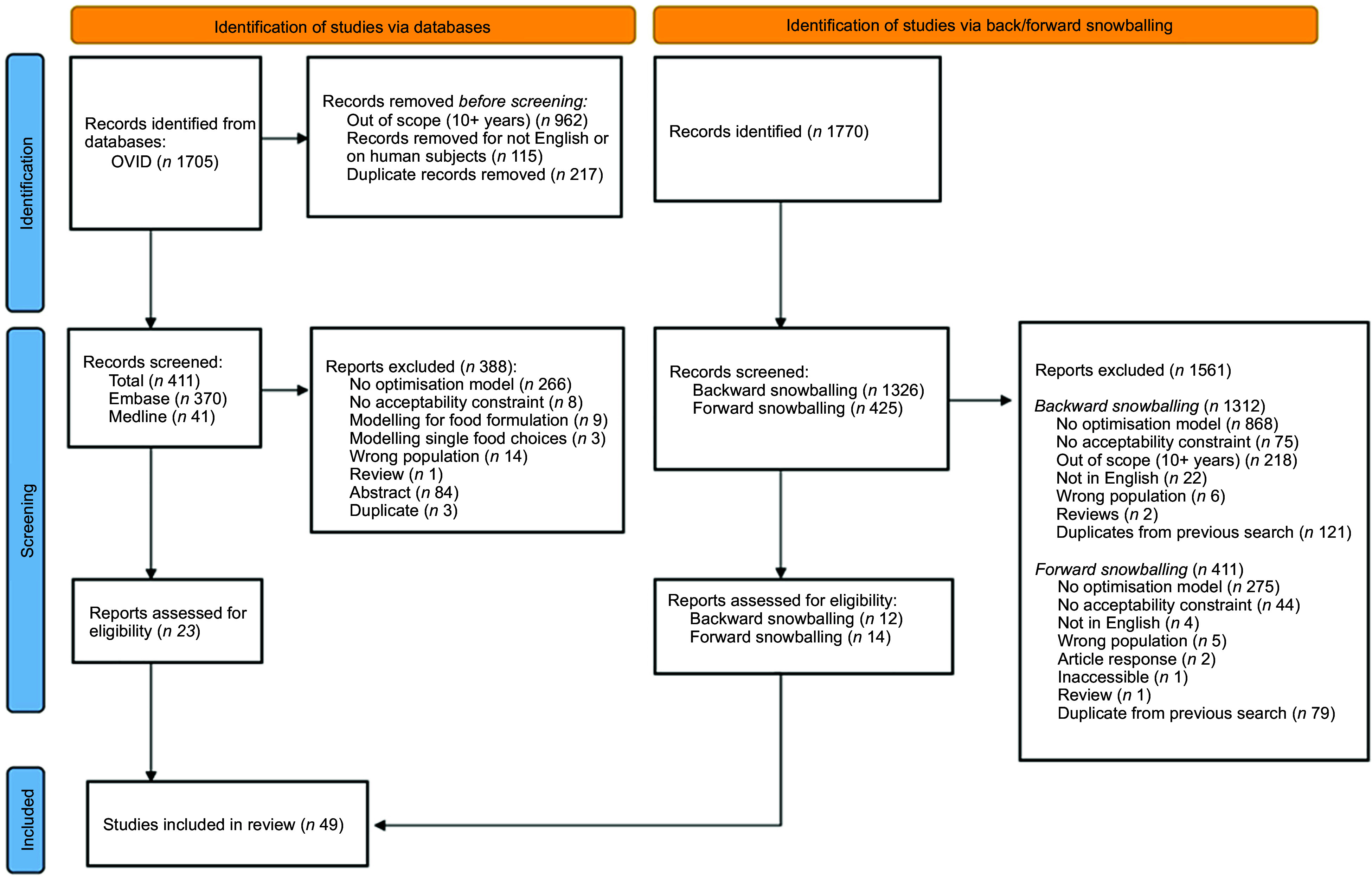
Figure 1 long description. PRISMA flow diagram demonstrating the process of the review and identification of eligible studies meeting criteria outlined in PICO criteria (see online supplementary material, Supplemental Tables S1, S2, S3) (from http://www.prisma-statement.org/).

A hybrid search strategy was implemented based on the definition outlined by Wohlin *et al.* ^(45)^, incorporating at least two systematic approaches to searching for relevant literature (Figure 1). The electronic database OVID (Embase, Medline) was searched for publications containing the identified search terms: ‘diet’, ‘diets’, ‘dietary’, ‘model(l)ing’, ‘optimis(z)ation’, ‘linear programming’, ‘acceptabl*’ dating from 2013 to June 2025 (see online supplementary material, Supplemental Tables S1, S2 and S3). The search terms were identified through an iterative process and were chosen to align with the research aim. The list of articles was extended using the backwards and forwards snowball search approach described by Wohlin^(46)^. Backward snowballing refers to the process of identifying references of interest from the initial set of papers, and twelve papers were included based on the inclusion criteria. Conversely, forward snowballing refers to the process of examining references citing the initial set of papers. Web of Science (Clarivate) was used to conduct the forward snowballing search in June 2023. Citations were downloaded and assessed based on the inclusion criteria, resulting in an additional fourteen papers. As no health outcomes were involved in this review, the protocol was not registered in the PROSPERO dataset.

### Data screening and extraction

C.B. independently screened all records for eligibility based on title/abstract, followed by a full-text screening. M.A.M independently and randomly screened eligibility of 10 % of the total records based on title and abstract. This resulted in a 94 % agreement rate on eligibility. The 6 % of studies, representing less than 1 % of total papers screened, that were not in agreement were discussed between C.B., M.A.M and B.dR.

A data extraction table was developed by C.B. to extract the data from each of the identified studies, including geographic location, data sources, types of dietary data, data of dietary data collected, models, objective functions, constraints (nutrition, economic, environmental and acceptability), where a definition was included, the definition used, for example quotes and key findings. The intended audience for the optimisations was also extracted following the Gazan *et al.* ^(10)^ definition of ‘population-based optimisation’ when applied to whole populations or subgroups and ‘individual-based optimisation’ when applied to individuals within a population. The diets currently consumed by the intended audience were identified as the observed diet, whereas the diet from the model was identified as the optimised diet.

### Data synthesis and analysis

How acceptability in diet modelling is defined and subsequently applied is likely to be influenced by the researcher’s knowledge and prior beliefs on commonly accepted dietary patterns^(47)^. Therefore, data on frameworks or approaches defining acceptability were extracted and interpreted using an adaptation of the three paths to food choice frameworks defined by Sobal and Bisogni^(41)^ (see online supplementary material, Supplemental Table S4). Briefly, *translation* refers to the use and/or adaptation of an existing perspective or framework; *deduction* represents the approach whereby experts use their own expertise, experiences and observations to develop new models or definitions; and *induction* concerns the use of information from a target audience to ground the models or frameworks in their perspectives. The extracted data were narratively synthesised^(48,49)^ and were weighted equally regardless of type and quality of study.

Qualitative data illustrating definitions and practical applications of acceptability were analysed using a *reflexive thematic analysis* approach proposed by Braun and Clark^(50)^. This six-step approach to descriptive theme development allows for the interpretation of the data and does not aim for an accurate nor replicable summary of the data. Rather, it aims to provide an interpretation grounded in the data through the lens of the researcher^(51)^. Papers were annotated and analysed for important meanings and understandings related to definitions of acceptability and the use of models, objective functions and constraints for acceptable outcomes. Papers were re-read a minimum of three times and quotes extracted. Key points were collated into categories and overarching themes, followed by a distillation into final themes and sub-themes (see online supplementary material, Supplemental Tables S5 and S6 for an overview of the six-step approach and the evolutionary process of final themes and sub-themes).

Lastly, data on the impacts on diet structure, nutrition, cost and environmental impact were extracted from the studies, where this was possible, either from the papers themselves or through the supplementary materials. It is important to note that the aim with this was not to compare the outputs of the models across the studies with each other since acceptability is context-dependent^(10,18)^, it was rather to provide a narrative synthesis of the overarching outcomes.

## Results

The initial search (May 2023) resulted in 808 studies. After removing out-of-scope and duplicated studies based on the <>PICOS criteria (Table 1), a total of 340 articles were thoroughly assessed. The second (May 2023–April 2024) and third search (April 2024–June 2025) resulted in 897 and 1008 studies, respectively. After removing studies that were out-of-scope, and duplicates, seventy-one and eighty-seven studies were included for assessment. The hybrid search strategy yielded a total of forty-nine studies; the digital database search yielded twenty-three studies, while the backward and forward snowballing approaches yielded twenty-six studies (Figure 1). The overview of studies included in the review can be found in Table 2.

**Table 2.** General characteristics of studies included in this systematic review. *Difference in years between dietary data collected and publication year Table 2 long description.

Study, Country	Audience	Dataset	Dietary data collection method	Number of foods	Type of food data	Year of dietary data collection	Difference in years*	Output
Kesse-Guyot *et al.* ^(16)^, France	Population	National survey	24-h recall	264	Observed diet	2014	8	Diet
Seconda *et al.* ^(52)^, France	Individual	National survey	24-h recall	239	Food items	2014	7	Diet
Masino *et al.* ^(53)^, Germany	Population	National survey	24-h recall	657	Food items	2007	16	Diet
Brouzes *et al.* ^(54)^, Egypt	Population	Local survey	4-day food diary	11	Food groups	2017	4	Diet
Broekema *et al.* ^(15)^, The Netherlands	Population	National survey	24-h recall	207	Food items and diet	2010	10	Diet
Chaudhary & Krishna ^(55)^, 152 countries	Population	Food balance sheet	Food balance sheet	221	Food items	2011	8	Diet
Sugimoto *et al.* ^(56)^, Japan	Individual	Local survey	4-day food diary	2229	Food items	2013	9	Diet
Verly-Jr *et al.* ^(57)^, Brazil	Population	National survey	2-day food diary	102	Food groups	2009	13	Diet
Kesse-Guyot *et al.* ^(58)^, France	Population	National survey	24-h recall	264	Food items	2014	7	Diet
Yin *et al.* ^(59)^, China	Population	National statistics	National statistics	13	Food groups	2017	3	Diet
Mariotti *et al.* ^(60)^, France	Population	National survey	7-day food diary	32	Food groups	2007	14	Dietary pattern
Kanellopoulos *et al.* ^(61)^, The Netherlands	Individual	Cohort study	Food frequency questionnaire	Not clear	Observed diet	2013	7	Diet
Yin *et al.* ^(34)^, China	Population	National statistics	National statistics	17	Food items	2017	4	Diet
Song *et al.* ^(62)^, China	Population	National survey	3-day food diary	1950	Food items and groups	2011	6	Diet
Perignon *et al.* ^(63)^, France	Population	National survey	7-day food diary	402	Food items and groups	2007	9	Diet
Verly-Jr *et al.* ^(64)^, Brazil	Population	National survey	Combination (24-h recall, 2-day food diary)	102	Food items	2009	11	Diet
Verly-Jr *et al.* ^(65)^, Brazil	Population	National survey	Combination (24-h recall, 2-day food diary)	102	Food items	2018	3	Diet
Thompson *et al.* ^(66)^, Spain, France, Sweden	Population	National survey	Combination (7-day food diary, 2-day food diary)	151	Food items	2007	6	Diet
Horgan *et al.* ^(14)^, United Kingdom	Individual	National survey	4-day food diary	134	Food groups	2011	5	Diet
Maillot *et al.* ^(67)^, France	Individual	National survey	7-day food diary	1314	Food items	2007	10	Diet
Wilson *et al.* ^(22)^, New Zealand	Population	Other source	Combination (Food Price Index, Supermarket data)	76	Food items	2009	4	Diet
Vieux *et al.* ^(18)^, France, United Kingdom, Finland, Sweden	Population	National survey	Combination (48-h recall, 4-day, 3-day, 7-day food diary)	151	Food items	2012	6	Diet
Tyszler *et al.* ^(32)^, France	Population	National survey	24-h recall	207	Food items	2010	6	Diet
Dussiot *et al.* ^(68)^, France	Population	National survey	24-h recall	45	Food groups	2015	7	Diet
Mertens *et al.* ^(69)^, Denmark Czech Republic, Italy, France	Population	National survey	Combination (24-h, 3-day, 7-day food diary)	22	Food items	2008	12	Diet
van Dooren & Aiking ^(70)^, The Netherlands	Population	National survey	24-h recall	206	Food items	2010	5	Diet
dos Santos *et al.* ^(71)^, Brazil	Population	National survey	2-day food diary	68	Food items	2009	9	Diet
Heerschop *et al.* ^(20)^, The Netherlands	Population	National survey	24-h recall	59	Food groups	2016	7	Diet
Sobhani *et al.* ^(72)^, Iran	Population	Food balance sheet	Food balance sheet	194	Food items	2018	4	Diet
Rocabois *et al.* ^(73)^, France	Both	National survey	7-day food diary	207	Observed diet	2007	15	Diet
Mazac *et al.* ^(74)^, 22 European countries	Population	National survey	Not clear	124	Observed diet	2013	9	Diet
Gazan *et al.* ^(75)^, France	Individual	National survey	7-day food diary	212	Food items	2007	15	Diet
Tompa *et al.* ^(76)^, Hungary	Population	National survey, Food balance sheet, National statistics	3-day food diary	53	Observed diet	2014	8	Diet
Benvenuti *et al.* ^(77)^, Italy	Population	National survey	Baseline menu	142	Dishes	Not clear	Not clear	Menu
Eini-Zinab *et al.* ^(78)^, Iran	Population	National survey	Not clear	194	Food items	2018	3	Diet
Grasso *et al.* ^(79)^, The Netherlands	Population	Cohort study	Food frequency questionnaire	254	Food items	2015	6	Diet
Maillot & Darmon ^(80)^, France	Individual	National survey	7-day food diary	206	Food items	2007	13	Diet
Lucas *et al.* ^(81)^, United Kingdom	Population	Food balance sheet	Food balance sheet	326	Food items	2011	10	Diet
Chaudhary & Krishna ^(82)^, India	Population	National survey	30-day dietary recall	111	Food items	2012	9	Diet
Abejon *et al.* ^(83)^, Spain	Population	National survey	Not clear	Not clear	Observed diet	2018	2	Diet
Barre *et al.* ^(84)^, France	Population	National survey	7-day food diary	402	Food items	2007	11	Diet
Green *et al.* ^(23)^, United Kingdom	Population	National survey	4-day food diary	42	Food groups	2011	4	Diet
Eustachio Colombo *et al.* ^(85)^, Sweden	Population	National survey	4-day food diary	24	Food groups	2011	13	Diet
Liu *et al.* ^(86)^, 181 countries	Population	Food balance sheet	Food balance sheet	10	Food groups	2020	4	Diet
Salome *et al.* ^(87)^, France	Population	Cross-sectional survey	Combination (24-h recall, portion size estimation)	1592	Food groups	2015	8	Diet
Irz *et al.* ^(47)^, Finland	Population	National survey	24-h recall	Not clear	Food groups	2017	7	Diet
Kesse-Guyot *et al.* ^(88)^, France	Population	Cross-sectional survey	Combination (24-h recall, portion size estimation)	15,92	Food groups	2015	8	Diet
Verly-Jr *et al.* ^(44)^, Brazil	Population	National survey	2-day food diary	110	Food items	2009	10	Diet
Verly-Jr *et al.* ^(89)^, Brazil	Population	National survey	2-day food diary	123	Food groups	2009	12	Diet

Defining acceptability, to a degree, was done by thirty-six studies, but few clearly stated, or provided further details, on their definition. For example, they may acknowledge the use of ‘cultural acceptability’, yet did not describe what this referred to (e.g. inclusion/exclusion of food items and diet structure). Thirteen studies mentioned acceptability as part of their model, yet provided no definition. Definitions of acceptability varied considerably across the studies, with six themes emerging (Table 3), of which the two most common themes are highlighted below. While these themes are presented as separate themes, several of them are interconnected, and several studies utilised multiple themes within the same study.

**Table 3.** Overarching themes used to define acceptability Table 3 long description.

Themes	Description	Studies
Culture	Based on cultural, social, traditional or local factors for the specific audience of the modelled diet.	(14,18,20,34,47,53,55–59,61,63–66,69–72, 75–78,82,84–90)
Familiar	The extent to which the output includes food items, groups or patterns that are already known, similar, recognised or commonly consumed.	(15,16,20,22,23,32,34,44,52–71,73,75,76,78–87)
Feasible	Whether the output realistically can be accessed, prepared, afforded, included in daily life given the context.	(15,18,20,32,44,55,56,62,69,77)
Theoretical	Acknowledging the conceptual nature of acceptability with data and assumptions made.	(44,80,87)
Context-specific	Based on the audience, model, type of data, constraints, which will change between studies and scenarios.	(15,66,72,77,82)
Palatable	Whether the food or diet has the necessary taste or texture.	^(70)^

Cultural relevance was the most common interpretation of acceptability and as such a core condition for acceptability in dietary optimisation. This interpretation aims to ensure that the diets developed align with cultural, social, traditional or local values, practices and food identities for the specific audience of the modelled diet.

*‘Modelling diets based on minimising the change to an individual’s current dietary intake allows individual food choices and habits, which are often driven by preferences, cultures or circumstance, to be accommodated’. (page 2)* ^(14)^

Food items or patterns either not reflective of average population consumption or recorded individual consumption are assumed unacceptable, regardless of their potential for consumption.

Familiarity was also mentioned as an important aspect of acceptability, defining acceptable outcomes as those that are already known, recognised or commonly consumed by the audience for the modelled outcome. This included both the types of food items, groups and dietary patterns.

*‘…the assumption that similar diets are more likely to be accepted by the majority of the population than more extreme diets are’. (page 703)* ^(32)^

The practical application of acceptability was synthesised across a framework of dietary optimisation modelling (Table 4) and detailed in Table 5. The first element of a model is the data input; here, acceptability was applied through context-specific foods, the availability of certain types of foods and the degree of popularity or familiarity of the foods. <>The most common method of dietary data collection was through national surveys (e.g. National Dietary Nutrition Survey in the UK or INCA2 survey from France) (*n* 35), followed by food balance sheets (*n* 4), local surveys (*n* 2), national statistics (*n* 2), cohort studies (*n* 2) and cross-sectional studies (*n* 2). One study used food item data from a combination of national average data and online supermarket data (see online supplementary material, Supplemental Figure S1). The methodological approaches to collect dietary data varied across the studies. The majority were based on 24-h recalls (*n* 11), followed by 7-day food diaries (*n* 7), a combination of methodologies (e.g. 24-h recall and 7-day food diary) (*n* 7), 4-day food diaries (*n* 5), 2-day food diaries (*n* 4), food balance sheets (*n* 4), unclear methodology (*n* 3), 3-day food diaries (*n* 2), food frequency questionnaires (*n* 2), national statistics (*n* 2), 30-day recall (*n* 1) and baseline menu (*n* 1) (see online supplementary material, Supplemental Figure S2). The number of food items or groups used as input to the models varied greatly between the studies, ranging from as few as ten food groups to as many as 1950 food items, with the higher number of food variables not typically used in individual-based optimisation. Three studies did not provide detail on the number of food items or groups used for the model. The difference in years between the collection of the dietary data and the year of publication was on average eight (see online supplementary material, Supplemental Figure S3). European countries had the largest representation (*n* 41) in the collection of data (see online supplementary material, Supplemental Figure S4).

**Table 4.** Thematic overview of practical applications of acceptability across stages of optimisation Table 4 long description.

	Sub-themes	Description	Studies
Data inputs			
Acceptable diets use…	Context-specific dietary sources	Includes the type of food, number of food items or groups, the source of the dietary data, which all influence the outcome of the model.	(52,66,70,72,77,82)
	Popular/familiar foods	Based on what the majority in the population eat, often limiting foods that are occasionally/never eaten.	(32,54,60,67,74,76,85)
	Cultural or religiously relevant foods	Based on the expert knowledge of the culture or religion or foods reported from data sources.	(66,70)
	Available foods	Assuming that all foods included are available to the audience at the time of the model.	(86)
	Demographic information	Includes education, income level, nutritional requirements specific to individuals or population groups.	(16,44,47,52,57,61,64,65,67,69,73,79,89)
Model elements			
Acceptable diets consider…	The quantity and quality of change	Includes the degree of the changes from baseline to optimised diet (small, large, progressive) and what type or foods are removed from the optimised diet.	(14,52–54,56–58,64,68,70,73,74,77)
	The audience	Based on the individual or population approach, which helps to account for the relation between the diet and audiences’ preferences.	(14,15,47,52,56,69,73,78,79,85)
	Boundaries	Used to constrain the values of the food items/groups towards certain assumptions.	(14,16,18,22,44,47,52,55,57,62–66,68,71,72,74–76,79–82,84–89,91)
	Substitutability	Assumes that certain food items or groups are considered more interchangeable than others.	(23,60,68)
	Quantity	Includes the total amount of food (e.g. grams or kcals) and individual food items or groups.	(18,44,60,81,82,84,85)
	Diversity/variety	Assumes that more food items or groups in the modelled diet are more acceptable.	(22,62,77)
	Elasticity	Based on price elasticity of demand, a measure of sensitivity to price changes. Minimising changes weighted by price elasticity to ensure sensitivity of food.	(23,59)
	Meal or dietary patterns	Based on dietary or meal patterns generally seen or recorded in the population.	(22,54,66,72)
Outputs			
Acceptable diets account for …	Output type	Either it is a single-day diet, a week diet, a dietary pattern or a menu.	(60,66,70,77)
	Evaluation	Contextualising the model outputs with literature (e.g. surveys, interviews, testing)	(56,59,69)

**Table 5.** Overview of optimisation elements (model, objective function, constraints and acceptability constraints) Table 5 long description.

Study	Model	Objective function	Constraints	Acceptability constraint(s)			
				First	Second	Third	Fourth
Kesse-Guyot *et al.* ^(16)^	Non-linear multistart	Minimise deviation from observed diet	Nutrition: DRV*; healthiness of diet scores (PANDIET and PNNS-GS2) Economic: Price of organic *v*. conventional foods (EUR) Environment: GHGE^†^ (kg of CO_2_eq); cumulative energy demand (MJ); land occupation (m^2^)	Boundaries (upper: 95th; lower: 5th)	NA	NA	NA
Seconda *et al.* ^(52)^	Hierarchical model with linear functions	Minimise deviation from observed diet	Nutrition: DRV*; healthiness of diet scores (PANDIET and PNNS-GS2) Economic: Price of organic *v*. conventional foods (EUR) Environment: GHGE^†^ (kg of CO_2_eq); cumulative energy demand (MJ); land occupation (m^2^)	Boundaries (upper: 95th; lower: 0)	NA	NA	NA
Masino *et al.* ^(53)^	Linear	Minimise total relative deviation	Nutrition: DRV*; FBDG^‡^ Economic: Price of diet (EUR) Environment: Upper limit of GHGE^†^ (kg of CO_2_eq) based on Intergovernmental Panel on Climate Change (IPCC)	Diversifying foods	Limit relative deviation (–50 % to +210 % or +260 %)	NA	NA
Brouzes *et al.* ^(54)^	Combination (Goal and linear programming)	NA	Nutrition: DRV* Economic: Price of food items (EGP)	Observed frequency of servings	NA	NA	NA
Broekema *et al.* ^(15)^	Quadratic	Minimise deviation from observed diet	Nutrition: DRV*; FBDG^‡^ Environment: Three GHGE^†^ targets (GHGE per person per day) with varying degree of strictness: low (2·5 kg CO2eq pppd), 2030 (2·04 kg CO2eq pppd) to 2050 (1·11 kg CO2eq pppd) based on IPCC 1·5-degree study	Limit deviation of total weight of diet between 33–150 % grams of food groups	NA	NA	NA
Chaudhary & Krishna ^(55)^	Non-linear	Minimise deviation from observed diet	Nutrition: DRV* Environment: GHGE^†^ (g CO_2_eq/cap/d), freshwater (L/cap/d), land (m^2^/cap/d), nitrogen (gN/cap/d), phosphorus (gP/cap/d) use	Limit deviation of total weight of diet between 80 – 120 % of observed diet	Boundaries (upper: 95th, lower: 0·1)	Constraint on individual foods or food groups	Constraint on individual foods or food groups
Sugimoto *et al.* ^(56)^	Data envelope analysis	Minimise deviation from observed diet	NA	NA	NA	NA	NA
Verly-Jr *et al.* ^(57)^	Linear	Minimise deviation from observed diet	Nutrition: WHO NCD^§^ prevention guidelines; vitamin and mineral requirements Economic: Cost (BRL) Environment: Stepwise reduction of GHGE^†^ (kg CO_2_eq) by 10 %	Boundaries (strict, lower: 5th/mean intake; flexible lower: 0/mean intake; upper: mean intake)	Constraint on individual food or food groups	NA	NA
Kesse-Guyot *et al.* ^(58)^	Quadratic	Minimise deviation from observed diet	Nutrition: DRV* Environment: GHGE^†^ (kg of CO_2_eq); cumulative energy demand (MJ); land occupation (m_2_); co-production factor for ruminant products (milk and meat)	Boundaries (upper: 95th)	Constraint on food groups	NA	NA
Yin *et al.* ^(59)^	Multi-objective	Minimise deviation from observed diet	Nutrition: DRV*	NA	NA	NA	NA
Mariotti *et al.* ^(60)^	Multi-objective	Minimise deviation from observed diet	Nutrition: DRV*; max limits on food groups associated with increased risk of NCD^§^; contaminant constraints for certain nutrients	Boundaries (upper: 95th)	Limit deviation to remain within range of observed intake	NA	NA
Kanellopoulos *et al.* ^(61)^	Data envelope analysis	NA	Nutrition: NRD^||^ 9·3	NA	NA	NA	NA
Yin *et al.* ^(34)^	Multi-objective	Minimise deviation from observed diet	Nutrition: DRV* Environment: Carbon footprint (GHGE^†^) (kg), water footprint (m^3^), ecological footprint (m^2^)	Limit deviation on food items and groups	Limit deviation of total weight of diet between 80–120 %	NA	NA
Song *et al.* ^(62)^	Linear	Minimise GHGE	Nutrition: DRV* Environment: Carbon footprint (COe2)	Boundaries (upper: 99^th^, lower: 50 % of observed intake)	Constrain on individual foods or food groups (specific foods kept at constant)	NA	NA
Perignon *et al.* ^(63)^	Linear	Minimise deviation from observed diet	Nutrition: DRV* Economic: Food prices (EUR) Environment: Increasingly stringent reductions of GHGE^†^ by 10 %	Boundaries (upper: 120 %, lower: 80 %)	NA	NA	NA
Verly-Jr *et al.* ^(64)^	Linear	Minimise total cost	Nutrition: DRV* Economic: Food prices (BRL)	Boundaries (vary progressively based on observed mean intake for each geographic-economic strata)	Constraint on food group (portion size)	NA	NA
Verly-Jr *et al.* ^(65)^	Linear	Minimise deviation from observed diet	Nutrition: DRV*; FBDG^‡^ (minimum fruit and vegetables); NOVA classification to restrict certain foods	Boundaries (upper: vary by 5g for food items, food group mean consumption)	NA	NA	NA
Thompson *et al.* ^(66)^	Linear	LiveWell model – Minimise sum of absolute deviation from average individual food intake	Nutrition: DRV*; FBDG^‡^; portion sizes Economic: Food prices (EUR)	Boundaries (60–80 % of current consumption, changed per country)	Boundaries (popular foods could increase by 4·5 times, not fall below 30 % of average value or 5 g/d; other foods allowed to increase by 3·3 times)	Boundaries (unpopular foods limited to no more than twice current average consumption)	Maximum portion size per individual food
Horgan *et al.* ^(14)^	Linear	Minimise deviation from observed diet	Nutrition: DRV* (Model 1 and 2) Environment: GHGE^†^ target (kg CO_2_eq/d (Model 2)	Boundaries derived from restricting change in weight to food items up to ≤ 50 %, if insufficient then maximum ±100 %	NA	NA	NA
Maillot *et al.* ^(67)^	Linear	Minimise deviation from observed diet; Minimise reduction of repertoire foods	Nutrition: DRV*, total diet weight (g/d) Economic: Cost of diet (EUR)	Constraint on maximum amount of food items and groups	NA	NA	NA
Wilson *et al.* ^(22)^	Linear	Various scenarios minimise cost and minimise GHGE	Nutrition: DRV* Economic: Cost of diet (NZD) Environment: GHGE^†^ (kg CO_2_-eq)	Boundaries (upper: 95 % of SI^¶^, lower: 95 % SI^¶^)	Familiar meals	NA	NA
Vieux *et al.* ^(18)^	Linear	Minimise deviation from observed diet	Nutrition: DRV* Environment: Stepwise (10 %) reduction of GHGE^†^ (g CO_2_-eq/d)	Limit deviation of total food weight ±20 %	Boundaries (upper: 90th, lower: 10th)	Solid-liquid ratio	Constraint on individual foods items or groups
Tyszler *et al.* ^(32)^	Linear	Minimise deviation from observed diet	Nutrition: DRV* Environment: GHGE^†^ (kg CO_2_eq/kg), fossil energy use (MJ/kg), land occupation (m^2^ × year/kg)	Penalty score	NA	NA	NA
Dussiot *et al.* ^(68)^	Non-linear multi-objective	Minimise deviation from observed diet	Nutrition: DRV*; FBDG^‡^ Environment: GHGE^†^ (kg CO_2_eq/kg)	Boundaries (upper: 95th, lower: 5th)	Constraint on individual foods or food groups (FBDG^‡^)	Substitutable food groups	NA
Mertens *et al.* ^(69)^	Data envelope analysis	1.Minimise deviation from observed diet 2.Maximise nutrient density 3.Minimise GHGE	Nutrition: FBDG^‡^, NRD^||^ 15. Environment: GHGE^†^ (kg CO_2_eq/kg)	Minimum deviation (MINDV)	Diet similarity index	Non-zero intakes	Caps on foods /nutrients when no additional benefit achieved
van Dooren & Aiking ^(70)^	Linear	Minimise absolute deviation	Nutrition: Health Score Environment: Combined GHGE^†^-land use score	NA	NA	NA	NA
dos Santos *et al.* ^(71)^	Linear	Minimise deviation from observed diet	Nutrition: EAR**	Boundaries (upper: 70th, lower: 10th)	NA	NA	NA
Heerschop *et al.* ^(20)^	Data envelope analysis	1.Minimise GHGE 2.MaximisingDHD15^††^	Environment: GHGE^†^ (kg CO_2_eq/2000 kcal)	Limit deviation (33 %)	NA	NA	NA
Sobhani *et al.* ^(72)^	Combination (linear and goal programming)	4 separate LP models (maximise NRF Index, minimise cost, water and carbon footprint) GP model combined all 5 objectives	Nutrition: DRV*, FBDG^‡^ Environment: Carbon (kg/d), water (m^3^/d), land (m^3^/d) footprint	Serving size	Boundaries (50 % lower or higher than observed intake)	NA	NA
Rocabois *et al.* ^(73)^	Other (IBDO and Individual Die Including Global Objectives Optimization)	IBDO – Minimise % deviation per individual Individual Die Including Global Objectives Optimization – Minimise sum of individual deviations across population	Nutrition: MAR^‡‡^, sed^§§^, MER^||||^ Environment: GHGE^†^ (g CO_2_eq/d)	NA	NA	NA	NA
Mazac *et al.* ^(74)^	Linear	Minimise environmental impact (GWP^¶¶^, LU, water use (WU))	Nutrition: DRV* Environment: GWP^¶¶^ (kg CO_2_eq), land use (m^2^ arable land equivalent), WU (m^3^)	Boundaries (upper: 95th, lower: 5th)	NA	NA	NA
Gazan *et al.* ^(75)^	Multi-objective	Minimise sum of deviation from observed diet	Nutrition: Energy intake +/–1 %; DRV*; FBDG^‡^ (limit of fish) Environment: 30 % reduction in GHGE^†^ (g CO_2_eq/d)	Boundaries (upper: 95th, lower: 5th)	NA	NA	NA
Tompa *et al.* ^(76)^	Linear	Minimise deviation from observed diet	Nutrition: RDI*** Environment: Water footprint (l/d/capita)	Boundaries (upper: 90th, lower: 10th)	Constrain on individual foods items or groups	NA	NA
Benvenuti *et al.* ^(77)^	Multi-objective	Minimise carbon and water footprint and cost	Nutrition: DRV* Environment: GHGE^†^ (g of CO_2_eq), water footprint (litres)	Repetition of dishes	Limit repetition of ingredients	NA	NA
Eini-Zinab *et al.* ^(78)^	Combination (LP and GP)	4 separate LP models (maximise NRF, minimise cost, water and carbon footprint) GP minimise total deviation from LP values	Nutrition: RDI***, WHO salt recommendation, FBDG Economic: Cost (RI) Environment: Carbon footprint (g CO_2_), water footprint (m^3^)	Boundaries (±50 % of usual intake)	NA	NA	NA
Grasso *et al.* ^(79)^	Quadratic	Minimise deviation from observed diet	Nutrition: DRV*; Food groups Environment: GHGE^†^ (kg CO_2_-eq/d)	Boundaries (upper: 95th)	NA	NA	NA
Maillot & Darmon ^(80)^	Linear	Minimise deviation from observed diet	Nutrition: FBDG^‡^, FBDG^‡^ with additional portion of dairy	Limit deviation of caloric total weight (120 % of observed diet)	Boundaries (upper: 95th or upper: 97·5th)	NA	NA
Lucas *et al.* ^(81)^	Linear	Minimise impact exceeding planetary boundaries	Nutrition: DRV* Economic: Price of food items (EUR) Environment: Absolute sustainability (9 planetary boundaries framework)	Boundaries (upper: 95th, lower: 10 %)	Limit total weight deviation by ±20 %	NA	NA
Chaudhary & Krishna ^(82)^	Non-linear	Minimise deviation from observed diet	Nutrition: DRV* Economic: Price of food items (INR) Environment: Absolute sustainability (9 planetary boundaries framework)	Boundaries (upper: 95th, lower: 0·1)	Limit total weight deviation between 0·8 and 1·5 times observed level	Constrain on individual food items or groups	No increase above 10 g/d for foods not currently consumed
Abejon *et al.* ^(83)^	Non-linear	1.Maximise NRD^||^ 9·3 2.Minimise GHGE 3.Minimise total cost of diet	Nutrition: DRV*	Limit deviation from observed diet	NA	NA	NA
Barre *et al.* ^(84)^	Combination (linear and non-linear)	Minimise deviation from observed diet	Nutrition: DRV* Economic: Diet cost (EUR) Environment: GHGE^†^ (reducing by at least 30 %); co-production of animal foods	Boundaries (upper: 90th)	Limit total diet quantity deviation between 80–120 % of observed diet quantity	NA	NA
Green *et al.* ^(23)^	Linear	Minimise deviation from observed diet	Nutrition: DRV*, WHO guidelines, fruit and vegetables Environment: GHGE^†^ (kg CO_2_eq per kg consumed food)	Price elasticities as weights	NA	NA	NA
Eustachio Colombo *et al.* ^(85)^	Linear	Minimisation of total relative deviation from the baseline diet	Nutrition: DRV*, FBDG^‡^ Environment: GHGE^†^ (g CO_2_eq per day)	Boundary (no more than 200 % from relative baseline weight)	NA	NA	NA
Liu *et al.* ^(86)^	Not clear	Minimise gap between nutrient levels and RDA inadequate in observed diet	Nutrition: MAR^‡‡^ Economic: Dietary cost Environment: GHGE^†^ (kg CO_2_eq); Freshwater withdrawals (L); Land use (m^2^)	Limit deviation by ±30 %	NA	NA	NA
Salome *et al.* ^(87)^	Non-linear multi-objective	Minimising deviation from current diet	Nutrition: DRV*, bioavailability (iron and zinc)	Boundaries (Upper: 95th, Lower: 5th)	Substitution foods grouped together	Boundary on individual foods (total intake of meat substitutes not above 99th percentile of initial meat consumption)	NA
Irz *et al.* ^(47)^	Quadratic	Minimise sum of squared relative deviations from observed average diet	Nutrition: DRV* Environment: GHGE^†^ (kg CO_2_eq/capita/d)	Boundary (Upper: 90th, Lower: 10th)	NA	NA	NA
Kesse-Guyot *et al.* ^(88)^	Non-linear multistart	Minimising deviation from current diet (Diet Departure), allowed for intra-group substitution	Nutrition: DRV*, FBDG^‡^ Environment: GHGE^†^ (kg CO_2_eq/d, blue water use (m^3^ water eq deprivation/d)	Boundaries (Upper: 99th)	NA	NA	NA
Verly-Jr *et al.* ^(44)^	Linear	Minimising deviation from current diet	Nutrition: RDI*** Economic: Cost of diet (BRL)	Boundaries (10th, 70th, 80th, 90th percentiles)	NA	NA	NA
Verly-Jr *et al.* ^(89)^	Linear	Minimise cost of diet	Nutrition: FBDG^‡^ (fruit and vegetables, sodium, sodium/potassium, calcium)	Boundaries (upper: 95th, lower: 5th)	Limit deviation progressively from averages by 5 g	NA	NA

*DRV – dietary reference values; ^†^GHGE – greenhouse gas emissions; ^‡^FBDG – Food based dietary guidelines; ^§^NCD – Noncommunicable diseases; ^||^NRD – Nutrient-Rich Diet score (15:3–15 nutrients to encourage, 3 nutrients to limit; 9:3–9 nutrients to encourage, 3 nutrients to limit); ^¶^SI – simulation interval; **EAR – Estimate average requirement; ^††^Dutch Health Diet Index 2015; ^‡‡^MAR – Mean adequacy ratio; ^§§^ sed – Solid energy density; ^||||^MER – Mean excess ratio; ^¶¶^GWP – Global warming potential; ***RDI – Recommended Dietary Intake Values.

**Figure 2.**
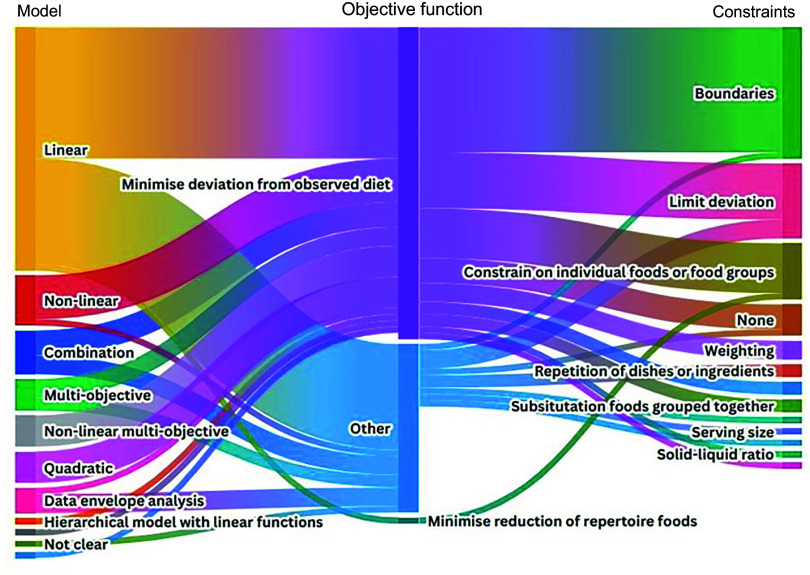
Figure 2 long description. Sankey diagram of links between models, objective function and constraints chosen to operationalise acceptability. Other refers to objective functions that do not relate to acceptability (i.e. nutritional score, environmental impact and cost). None refers to studies that do not use constraints specific to acceptability, and they may have used other constraints related to nutrition, environment or cost.

The second element involved the specific features included in the model, where acceptability was applied through the type of model, objective function, the audience (individual *v*. population approach), types of boundaries set, substitutability of foods and quantity of foods (Table 4). Of the forty-nine studies, forty-one took a population-based modelling approach, seven took an individual-based approach and one study combined both approaches. Methodologically, the most common model was LP (*n* 23), followed by quadratic programming (*n* 4), a combination of models such as goal and LP or linear and non-linear (*n* 5) and multi-objective programming also known as Pareto optimisation (*n* 4) (Table 5, see online supplementary material, Supplemental Figure S5). The outcomes of the optimisation studies were to model diets (*n* 46), dietary patterns (*n* 2) or menu (*n* 1). The most prevalent objective function was to minimise the changes from the observed or baseline diet, expenditure (*n* 31), with either no acceptability-related objectives (*n* 17) or a combination of two acceptability-related objectives (e.g. minimising deviation from diet and minimising reduction of repertoire foods) (*n* 1) (see online supplementary material, Supplemental Figures S6 and S7). Acceptability-related constraints were more widespread. The average number of acceptability constraints included in the models was one, with the most utilised constraint being the setting of boundaries (*n* 32), followed by limiting deviation (*n* 15) and constraining individual foods or food groups (*n* 12) (Figure 2).

The final element involved how acceptability of the optimised output was applied. This covers two themes, whether the optimised diet was created as a single-day diet, weekly diet or a menu and whether the optimised outputs were contextualised within grey or academic literature or tested through quantitative methods (e.g. survey, food waste assessment) or qualitatively (e.g. interviews). Similarly, in defining acceptability in Table 3, the themes are presented separately; however, many are interrelated and were used across studies.

Analysis of the approaches (see online supplementary material, Supplemental Table S8) employed by studies found that translation (the use or adaptation of existing perspectives) was the most commonly approach taken. This was either through the assumptions made (*n* 3)^(14,53,68)^, the definitions (*n* 6)^(16,34,53,70,73,76)^, the models (*n* 7)^(20,53,57,61,69,77,79)^ and the objective functions used (*n* 9)^(18,32,52,56,62,66,68,80,85)^. Deduction (the use of expertise, experiences or observations to develop new models or definitions) was primarily used to develop boundaries (*n* 18)^(16,18,44,52,57,58,60,63–66,68,71,72,75,86,88)^ and constraints (*n* 14)^(18,20,22,32,34,44,53,60,63,67,69,77,81,83)^. Lastly, the path of induction (applying data from citizens to ground models in their perspectives) was commonly used to decide on data to input into the model (*n* 49). In addition to the induction pathway, two studies also used the other pathways; one study used translation approach to identify foods from other studies^(22)^, while another used deduction to identify observations within the dietary data^(85)^.

Modelling for acceptability in dietary optimisation studies will have implications for the dietary solutions; however, the specific impacts were difficult to determine as most studies did not independently assess or compare their acceptability models, objective functions, boundaries or constraints with baseline diets or other optimised diets. While not the aim of the studies, the authors were able to deduce the impacts from results from eighteen studies (Table 6). Diets modelled for acceptability generally resulted in more plant-based compositions that included smaller amounts of animal-based products – particularly chicken, fish and eggs, and a complete to partial reduction in ruminant meats such as beef, compared to observed diets of the study population. Incorporating acceptability into diets did not drastically compromise the nutritional content of the modelled output and in the studies that measured acceptability resulted in increased overall health or nutritional score^(15,20,56,68)^ compared to observed diets, but not always compared to other models with stricter nutritional or environmental constraints^(68,69)^.

**Table 6.** Brief summary review of studies where practical application of acceptability could impact model outputs Table 6 long description.

Study, Country	Model output	Acceptability format*	Acceptability constraint impact on			
			Diet structure	Nutrition	Cost	Environmental impact
Masino *et al.* ^(53)^, Germany	Modify current diet	Objective function	Diets reduced by 9 food items for a total of 112 foods. Meat and meat products reduced slightly (89 g/d–60 g/d) for women and increased by 1 g/d and reduced 77 g for men, respectively. Pulses increased by around 13–15 g/d women and men respectively, and fish products reduced (8 g/d women and 10 g/d men).	NA	Reduced cost by 1·62 euros for men and 2·36 euros for women.	Significant drop (−32·5 %) for men and moderate (2·2 %) for women.
Brouzes *et al.* ^(54)^, Egypt	Combination (Modifying current diets, Food replacement, Balancing trade-offs)	Model, constraint	Servings per week of food groups vegetables (58 % more), yogurt (removed), fruit (54 %), tahini (92 %), Nile tilapia, red meat, liver, and eggs (∼100 %).	< 80 % of the iron constraint was not met, and all other nutrients reached or exceeded their constraints.	NA	NA
Broekema *et al.* ^(15)^, The Netherlands	Combination (Modifying current diets, Diet substitution)	Model, objective function, constraint	Meat (+14 g/d), cheese (+10 g/d), and unsaturated oils (+34 g/d) were higher than in the stricter environmental model. Legumes (2 g/d), soy foods, and tree nuts were lower.	Possible to meet dietary recommendations. B_12_ is higher by 2·57 mg compared to vegan or lacto-ovo-vegetarian, slightly lower than stricter environmental models and flexitarian diet.	NA	2·04 kg CO_2_-eq per person per day, similar to the strict environmental model, over 50 % change compared to the baseline diet.
Sugimoto *et al.* ^(56)^, Japan	Modifying current diets	Model, objective function	9 % (men) and 10 % (women) lower Diet similarity Index compared to the observed diet, although this was lower than the scenarios only modelling GHG reduction, NRF score and cost. Combining all four goals had the least deviation from an observed diet with 6 % (both).	8 % (men) and 10 % (women) higher Nutrient-Rich Food Index 15·3 Score than the observed diet.	6 % (men) and 2 % (women) less costly than observed diet.	13 % (men) and 10 % (women) lower diet-related greenhouse gas emissions compared to observed diet.
Verly-Jr *et al.* ^(57)^, Brazil	Modifying current diets	Constraints	Flexible constraints allowed for red meat removal when GHGE was reduced by 70 %, seafood/fish increased with a reduction of 40 % GHGE. Red meat halved in strict acceptability models at 30 % GHGE reduction. Changes between the two constraints were mainly around red meat, chicken and high fat salt sugar (HFSS) foods.	NA	Flexible constraints allowed for red meat removal when GHGE was reduced by 70 %, and seafood/fish increased when up to reduction of 40 % GHGE. Red meat halved in strict acceptability models at 30 % GHGE reduction. Changes between the two constraints were mainly around red meat, chicken and HFSS foods.	70 % reduction in GHGE from baseline compared to 30 % GHGE reduction with stricter acceptability constraints.
Verly-Jr *et al.* ^(65)^, Brazil	Combination (Modifying current diets, Food replacement)	Constraints	Fish, dairy products, fruits, vegetables, and vegetable oils didn’t increase as needed for nutrition, lower than flexible acceptability constraint.	Limiting nutrients, calcium, magnesium, vitamins D and E, and fibre. Iron and copper were met by 100 % of the geographic stratum.	NA	NA
Yin *et al.* ^(59)^, China	Combination (Balancing trade-offs, Modifying current diets, Diet substitution)	Objective function	The highest increase in vegetables (176·19g/d), beef reduced but by less than environmental and health (15·84 *v*. 5·07 g/d).	Calories were reduced, protein increased slightly, fat and carbohydrates were reduced.	NA	Increased carbon food by 0·35 kg, water food print (53·3 m^3^) and reduced ecological food print (m^2^) by 427·38.
Kanellopoulos *et al.* ^(61)^, The Netherlands	Modifying current diets	Model	Differed across age and gender groups (e.g. young males increased potatoes, milk, dairy), young females lower intakes of potatoes, higher intake of eggs. Fish increased for all groups except younger males and females. Lower intakes of bread, nuts, snacks, fats, oil, soups, pastry, cakes, sugar/sweets.	Overall improvement in nutrient intakes (reduction in sodium, saturated fat, added sugars) and increase in protein, fibre, calcium, iron, etc. Slight variations between gender and age groups.	NA	NA
Song *et al.* ^(62)^, China	Combination (Modifying current diet, Food replacement)	Constraints	36 % and 50 % lower meat and rice, 84–128 % increase in fruits for males and females, 19–31 % increase in yogurt for males and females. Higher intakes of maize and tubers.	NA	NA	2971 and 2799 cCO_2_e per day men and women, 15 % and 9 % reduction compared to the observed diet.
Horgan *et al.* ^(14)^, United Kingdom	Modifying current diets	Objective function, constraints	Greater changes needed for a sustainable diet than a healthy diet. More commonly added oily fish, white fish, nuts and seeds, less vegetables, fruit, teas and coffee.	Sodium and fibre limiting nutrients, all other nutrients met.	NA	About 1 kg less per day with all four constraints. A maximum 50 % increase and adding new foods had a large impact on reducing GHGE differed slightly by how many new items and worked for most participants.
Wilson *et al.* ^(22)^, New Zealand	Combination (Hypothetical diets, Diet Substitution, Balancing trade-offs)	Constraint	The number of food items increased to 19 compared to other models.	Increase in fruit and vegetables compared to optimisation models with just cost or GHGE (i.e. apples). Wholemeal bread, sausage, mince, sardines, and tuna were included in separate models. Total weight of foods consumed per day increased from 914 g to 1204–1658 g for the four acceptability models.	Higher cost of up to $6·75 /d compared to other low-cost optimised diets, lower than the average diet.	The highest GHGE up to 5·98 kg CO_2_eq/d but far lower than average emissions of 10·1kg CO_2_eq/d.
Dussiot *et al.* ^(68)^, France	Combination (Modifying current diets, balancing trade-offs)	Model, objective, constraint	Soft drinks included lower whole grain products, more refined grains (men), and lower amounts of fruit and vegetables, which differed slightly across men and females with different iron requirements.	Health scores increased for all males (0·17–1·04) but not as much as when acceptability was removed (2·16–2·80), a similar pattern for females. Bioavailable iron and zinc were better with flexible models and with acceptability constraints.	NA	NA
Mertens *et al.* ^(69)^, Denmark, Czech Republic, Italy and France	Combination (Modifying current diets, Balancing trade-offs)	Model, constraints	Higher calories than the observed diet (6–11 %), the acceptability model had more alcoholic and sweet beverages than other models. Animal-sourced foods remained similar to the observed diet (35 % of total weight), characterised by higher fish, eggs and total dairy.	Least improvement on nutrition inadequacies with the acceptability model. NRD15·3 increased for all countries with an acceptability model. Sodium and added sugar increased slightly from the observed diet in Denmark and Czech Republic	NA	Reduction in greenhouse gasses with acceptability model for all except women in Czech Republic.
Heerschop *et al.* ^(20)^, The Netherlands	Modifying current diets	Model	Higher amounts of fruits, vegetables, nuts, and legumes compared to observed diets, but this differed between subgroups and quartiles of meat consumers. Older women had little improvement because they were already eating more plant-based foods.	Iron and calcium are above EAR, and little difference between optimised and current diets; vitamin B_12_ increases but most drastically for older women.	NA	Average GHGE exceeded the planetary boundary of 2·04 kg CO_2_-eq by 2030 (3·5kg CO_2_eq).
Lucas *et al.* ^(81)^, United Kingdom	Combination (Hypothetical diets, Balancing trade-offs)	Objective function, constraints	Double intake in g of rice, wheat, corn, and others compared to baseline diet, about 100 g more of potatoes, reduced amount of fruits and vegetables, no increase in legumes, nuts, soy. Increase in fish/seafood but reduction in all other animal foods.	NA	£1·05 less than baseline diet.	Reduction in all planetary boundaries compared to the baseline diet has a similar impact to the least transgression model except for energy use, nitrogen, phosphorus, and land-system change.
Abejon *et al.* ^(83)^, Spain	Combination (Modifying current diets, Diet substitution)	Constraint	NA	Limiting nutrients included calcium, sodium, vitamin A, SFA and potassium for the different diets. With the increase in variety increase in NRD9·3 score	As a variation from the current diet increased up to 60 %, price was reduced for all dietary patterns; however, this change wasn’t similar. Mediterranean diet had a larger reduction.	GHGE was reduced with all planetary boundaries and was between 2 and 3 kg CO_2_eq/d.
Eustachio Colombo *et al.* ^(85)^, Sweden	Combination (Modifying current diet, Hypothetical diets)	Constraint	The total number of foods available was reduced by 266–249. The number of foods removed increased differed across clusters and average diet (Classic cluster has highest foods increase by 58).	NA	All three clusters have slightly lower costs (∼4 Swedish krona).	Clusters of dietary intake had similar CO2eq compared to using average population intake. The cluster around classic baseline diet has the highest reduction in CO2eq.
Verly-Jr *et al.* ^(44)^, Brazil	Modifying current diets	Objective function, constraint	Increases in fruit and vegetables were highest in the flexible model, including fruits, beans, fish, seafood, yogurt, and non-fat milk products.	Higher the flexibility, the higher the nutrient quality. Sodium was higher with rigorous acceptability constraints; all other nutrients were higher with the flexible acceptability constraint.	Moderate acceptability model has the highest increase in cost (R$6·7) compared to rigorous (R$6·24) and flexible (R$5·99).	NA

GHGE – greenhouse gas emissions; EAR – Estimate average requirement.

*Acceptability Format refers to how acceptability was applied in the optimisation. Model refers to the model used (e.g. linear, multi-objective), objective function refers to objective function used (e.g. minimise deviation and maximising scores), constraint refers to constraints used (e.g. boundaries, limiting deviation and diversifying foods).

## Discussion

In this review, we established that acceptability is not consistently defined across dietary optimisation studies, highlighting the need for a shared understanding of what is meant by an acceptable diet. Most studies described acceptability in terms of *cultural relevance* or *familiarity*. Other definitions included the terms *feasible, theoretical, personal, context-specific* and *palatable.* However, there was no common framework guiding these interpretations. The way acceptability was applied in practice also varied widely. Common approaches included minimising changes from existing dietary patterns through objective functions or setting upper and lower limits for certain foods. Other studies added constraints ensuring dietary variety and diversity, while some aimed to reflect real-world habits by contextualising modelled diets within ‘real-world’ settings.

Whilst most studies defined acceptability to some extent, few provided a clear or consistent definition and often had a considerable degree of subjectivity, in agreement with Heerschop *et al.* ^(20)^, Kanellopoulos *et al.* ^(61)^ and with Thompson *et al.* ^(66)^ view of it as a ‘nebulous’ term. Among the six themes identified in this review, the two most common interpretations of acceptable diets were those that emphasised cultural relevance, e.g. assuming that the foods, or combinations of foods, provided to the model are culturally appropriate, as noted by House *et al.* ^(28)^, and those that focus on familiarity, assuming that all foods included are generally acceptable to the population or individual^(22)^. These definitions often rely on broad assumptions. One key assumption is that available data accurately represent foods acceptable by the population^(10,47)^. Another is that food consumption patterns remain stable, regardless of factors such as life stage or changes in eating behaviours. However, this raises issues for foods only occasionally consumed in certain contexts, such as seafood, nuts and legumes^(69)^. In this review, we defined *acceptable diets* as those shaped by conditioned food preferences, social and cultural norms and established eating practices. Nevertheless, future research could employ methods such as Delphi panels focused on diet and food acceptability to develop a more comprehensive and nuanced understanding of this complex area^(92)^.

Diet optimisation approaches, including linear and quadratic programming, data envelopment analysis^(61)^ and individual-based frameworks such as Individual Die Including Global Objectives Optimization^(75)^, offer diverse approaches to incorporating acceptability. These models differ in how they address deviation from observed diets, whether they target individuals or populations and manage trade-offs between competing objectives. LP, the most commonly applied method, typically minimises total deviation without considering how changes are distributed across food items or groups, often resulting in large, concentrated changes in specific foods^(10,13)^. Within LP, some models minimise absolute deviation^(14,32)^, resulting in fewer but larger changes, while others minimise relative deviation, resulting in smaller, more proportional adjustments across the diet^(85)^. If smaller and widespread changes are considered more acceptable, minimising relative deviation may serve as a better proxy for diet acceptability. Quadratic programming further refines this by minimising the sum of squared deviations, spreading smaller changes across more foods, favouring small, incremental adjustments, which are often seen as more realistic and acceptable compare to LP^(79,93)^.

Model objects also differ in scope. Traditional models (e.g. LP) tend to optimise diets at the population level, assuming a single solution can be generalised across individuals in a population. However, this approach overlooks the fact that few people consume the average diet, as personal preferences, habits and context are highly individuals^(10)^. In contrast, individual-based models like Individual Die Including Global Objectives Optimization minimise dietary change at the individual level, while maintaining environmental goals at the population level^(73)^. Data envelope analysis presented by Kanellopoulos *et al.* ^(61)^ and applied by Mertens *et al.* ^(69)^ benchmarks solutions using linear combinations of observed diets, implicitly incorporating acceptability by not creating theoretical diets and staying close to the observed consumption patterns and maintaining food substitutability^(61)^. Multi-criteria models take a flexible approach by integrating acceptability (e.g. minimising dietary change) alongside objectives such as health, cost and environmental impact. This approach provides a spectrum of solutions rather than a single optimised diet^(34)^. Models have evolved from traditional LP approaches to more complex multi-criteria models that better integrate acceptability and model for individual preferences.

Setting lower and upper boundaries for food items and groups is a common method used to constrain dietary intake and minimise deviation from observed diets^(64)^. This approach helps limit the inclusion of unfamiliar foods and the exclusion of commonly consumed foods^(14)^, while also giving modellers the flexibility to refine dietary models^(66)^ in ways considered acceptable. Boundaries (minimum and maximum intake levels for each food) are calculated per item^(44,64)^; however, there is no consensus on how wide these ranges should be. Some studies apply percentile-based boundaries (e.g. 10th, 70th, 80th, 90th percentiles)^(44)^, which reflect population intake distributions and avoid arbitrary cut-offs, making them useful especially where intake varies widely. Others use percentages (e.g. 80 % of intake *v*. 120 % of intake)^(63)^, which are suited to reflect proportional changes relative to the observed diet. Using the 5th and 95th percentiles is often justified as these values encompass the central 90 % of intake, avoiding extremes that, while acceptable to outliers, may not suit the wider population. However, the suitability of these boundaries should depend on the quality of the data, how well the data represents the target population and the overall nutritional status. Broader boundary ranges are sometimes used to model a wider range of dietary patterns, increasing flexibility and the likelihood of generating diverse and acceptable outcomes. Using boundaries in dietary modelling provides an easy and flexible approach to incorporating acceptability; however, their application remains subjective, and instead, we need more standardised approaches that incorporate social and behavioural contexts.

Alternative approaches of incorporating acceptability constraints in diet optimisation include accounting for cooking and preparation methods, which are often culturally specific^(39)^. For example, a UK study found that individuals were most confident with simple techniques like boiling, microwaving and stir-frying, while less familiar with pressure cooking and sous vide methods^(94)^. Wilson *et al.* ^(22)^ introduced a ‘food skills constraint’ that limited diets to foods requiring minimal culinary skills, excluding items such as flour, lentils, semolina, couscous and dried peas. Taste is another critical factor influencing food acceptance^(95)^. Sustainable diets, which often reduce umami, salt, fat and bitterness^(96)^, may be less appealing to consumers. Developing constraints based on taste profiles or common flavour pairing could enhance diet acceptability. Donati *et al.* ^(97)^, although excluded from this review, proposed incorporating typical meal compositions to ensure culturally appropriate pairings (e.g. biscuits with tea/coffee) and avoid incompatible combinations (e.g. beef and chicken in the same meal). However, research remains limited on how these meal patterns might shift during the transition to sustainable diets. For instance, it is unclear whether increased consumption of certain proteins, like chicken, could influence preferences for specific carbohydrate sources, such as rice over bread or pasta. Dietary diversity has also been used as a constraint, thought there is no consensus on what constitutes an appropriate diverse diet in terms of the number of food items to include (see Table 2)^(98)^. In his review of LP for optimised diets, Van Dooren^(13)^ suggests that including a greater number of food items can enhance both dietary diversity and acceptability. While integrating acceptability into diet optimisation models improves their potential for real-world applications^(10)^, the true acceptability of modelled diets remain unknown without conducting empirical evaluations with the target population^(10,54,63)^. Only three studies included ad-hoc assessments of acceptability, using tools such as health and environmental distance scores^(70)^, the Diet Similarity Index^(56,69)^ or by contextualising findings within current trends like meat reduction and flexitarianism^(69)^. Other studies excluded from this review due to selection criteria (Table 1) offer valuable examples of empirical evaluation, including consumer satisfaction surveys, and contextualisation efforts^(11,99,100)^, though such interpretations may be influenced by the researcher’s assumptions and prevailing dietary norms^(47)^. Although assessing the impact of acceptability on model outcomes was beyond this review’s scope, we identified where its practical application could influence results. Incorporating acceptability can enhance diet quality, diversity and environmental outcomes, but often involves trade-offs between health, affordability and sustainability. This underscores the need for context-specific approaches and compromises based on population’s preferences^(66)^. Further research should explore how different acceptability applications affect model outputs, ideally through systematic comparisons within a single cohort to ensure consistency. Overall, including acceptability in optimisation models tends to improve nutritional quality from observed diet without significantly limiting reductions in greenhouse gas emissions.

Acceptability in diet optimisation has been described as involving ‘a substantial degree of subjectivity’^(61)^. In this review, we analysed how studies define acceptability using an adapted version of the three paths to food choice framework^(41)^ (see online supplementary material, Supplemental Figure S8). *Induction* (i.e. drawing directly from the public) was exclusively used for model inputs, highlighting missed opportunities to incorporate public perceptions and preferences more fully through the optimisation process. For example, Yin *et al.* ^(59)^ measured willingness to change meat consumption on a scale of 1–10, assuming that low willingness indicates unacceptability of certain dietary changes. *Translation* (i.e. adapting existing perspectives) was used in model design, objective functions and underlying assumptions taken, reflecting a reliance on the status quo and pointing to a gap for innovation. *Deduction* (i.e. use of expert judgement) was common when setting boundaries and constraints, underlining the subjective nature of current practices. This presents potential to integrate broader perspectives, such as convenience around food preparation^(65)^ or taste preferences through meal composition^(97)^. Behavioural indicators like intention to consume or attitudes may also be useful, especially when individual-based modelling is not possible. In such cases, sociocultural frameworks like Passim & Bennett’s classification, based on food use and frequency^(39)^ offer alternatives. Drawing from fields such as behavioural science, psychology, sociology, food science, user acceptability research and ethnographic studies may support more comprehensive approaches. Understanding how acceptability is currently defined reveals key research gaps and can guide future efforts to ensure diets optimised for nutrition, environmental impact and costs are also acceptable for their intended audiences.

Despite a broad search using a hybrid strategy, certain limitations are present. The snowballing method may have led to clustering around certain authors, potentially missing other relevant work, though this risk is minimal given the strong initial set and diminishing returns for each search step. Secondly, this review did not consider specific population groups, despite food acceptability varying by age, gender and other personal factors.

### Conclusion

To our knowledge, this is the first systematic review examining how dietary optimisation studies define and apply acceptability. While dietary optimisation is increasingly used to support transitions towards sustainable diets, acceptability remained less integrated than other dimensions such as nutrition, cost and environmental impact. Approaches like cultural contextualisation, minimising deviation and setting food item boundaries are common, and this review found considerable variation within these methods, as well as innovative approaches such as Individual Die Including Global Objectives Optimization and data envelope analysis. Echoing the sentiment expressed by Gazan *et al.* ^(10)^, who call for greater transparency and justification in modelling, we propose five recommendations: (1) clearly define acceptability using social and behavioural theory; (2) tailor input to the target audience using local or consumer data and population segmentation; (3) justify acceptability-related parameters with appropriate references; (4) assess the impact of acceptability across the optimisation process; and (5) contextualise outcomes through empirical testing or trend analyses.

## Supporting information

10.1017/S1368980026102717.sm001 Baungaard et al. supplementary material Baungaard et al. supplementary material

## Supplementary material

For supplementary material accompanying this paper visit https://doi.org/10.1017/S1368980026102717

## Table 1. Long description

The table presents the PICOS criteria used for inclusion and exclusion in a systematic review on dietary optimization studies. It includes five main categories: Population, Intervention, Comparator, Outcome, and Study Design. Each category lists specific criteria for inclusion and exclusion. The Population category specifies that the study must include adults aged 18 years and above, excluding individuals under 18, medical populations, and families with individuals under 18. The Intervention category requires dietary modeling studies that include acceptability as an objective function, constraint, or claimed modeled diet, published within the last 10 years from May 2013, excluding studies without mention of acceptability or published before 2013. The Comparator category is marked as not applicable. The Outcome category focuses on methods of designing and defining acceptability and its impact on dietary solutions, excluding studies without mention of acceptability in the model or contextualizing the model. The Study Design category includes dietary optimization studies published in English, excluding non-diet optimization studies, optimizations for animals, and non-English publications. The table is structured with five rows and three columns, providing a clear framework for the systematic review.

Navigate back to Table 1..

## Figure 1. Long description

The flowchart begins with the identification of studies via databases and back/forward snowballing. Records identified from databases include OVID with 1705 records, Embase with 411 records, and Medline with 41 records. Records removed before screening include those out of scope, not in English, and duplicates. Records screened from databases total 411, with exclusions for no optimization model, no acceptability constraint, modeling for food formulation, wrong population, reviews, abstracts, and duplicates. Reports assessed for eligibility number 23. Studies included in the review total 49. Records identified via back/forward snowballing number 1770, with 1326 from backward snowballing and 425 from forward snowballing. Reports excluded number 1561, with specific reasons for exclusion listed. Reports assessed for eligibility number 26, with 12 from backward snowballing and 14 from forward snowballing. The final studies included in the review total 49.

Navigate back to Figure 1..

## Table 2. Long description

The image presents a detailed flowchart outlining the search and selection process of studies for a systematic review. The initial search in May 2023 resulted in 808 studies. After removing out-of-scope and duplicated studies based on the PICOS criteria, 340 articles were thoroughly assessed. The second search, conducted between May 2023 and April 2024, yielded 897 studies, which were narrowed down to seventy-one studies after removing out-of-scope and duplicate studies. The third search, from April 2024 to June 2025, resulted in 1008 studies, with eighty-seven studies included for assessment after similar filtering. The hybrid search strategy produced a total of forty-nine studies, with twenty-three from digital database searches and twenty-six from backward and forward snowballing approaches. The overview of studies included in the review can be found in Table 2.

* Difference in years between dietary data collected and publication year.

Navigate back to Table 2..

## Table 3. Long description

A table with three columns and seven rows, comparing themes, descriptions, and studies related to dietary acceptability. The first column lists themes such as Culture, Familiar, Feasible, Theoretical, Context-specific, and Palatable. The second column provides descriptions for each theme, explaining factors like cultural influence, familiarity of food items, feasibility of access and preparation, theoretical acknowledgment, context-specific considerations, and palatability. The third column lists study references associated with each theme. Notable trends include the interconnectedness of themes and the use of multiple themes within single studies.

Navigate back to Table 3..

## Table 4. Long description

A table with three columns labeled Sub-themes, Description, and Studies, detailing the practical applications of acceptability in dietary optimization models. The table is divided into sections: Data inputs, Model elements, and Outputs. Data inputs include Acceptable diets use with sub-themes like Context-specific dietary sources, Popular/familiar foods, Cultural or religiously relevant foods, Available foods, and Demographic information. Model elements include Acceptable diets consider with sub-themes like The quantity and quality of change, The audience, Boundaries, Substitutability, Quantity, Diversity/variety, Elasticity, and Meal or dietary patterns. Outputs include Acceptable diets account for with sub-themes like Output type and Evaluation. Each sub-theme is described and associated with specific studies listed in the third column. The table provides a comprehensive overview of how acceptability is applied in different stages of dietary optimization modeling.

Navigate back to Table 4..

## Table 5. Long description

The diagram presents an overview of the practical application of acceptability in dietary optimisation modelling. It outlines the framework of dietary optimisation modelling and details the elements involved. The first element is the data input, which applies acceptability through context-specific foods, the availability of certain types of foods, and the degree of popularity or familiarity of the foods. The diagram lists various methods of dietary data collection, including national surveys, food balance sheets, local surveys, national statistics, cohort studies, and cross-sectional studies. It also mentions the use of food item data from national average data and online supermarket data. The methodological approaches to collect dietary data vary, with the majority based on 24-h recalls, followed by 7-day food diaries, combinations of methodologies, 4-day food diaries, 2-day food diaries, food balance sheets, unclear methodologies, 3-day food diaries, food frequency questionnaires, national statistics, 30-day recalls, and baseline menus. The number of food items or groups used as input to the models varies greatly, ranging from ten food groups to 1950 food items. The diagram also notes the average difference in years between the collection of the dietary data and the year of publication, which is eight years. European countries have the largest representation in the collection of data.

Navigate back to Table 5..

## Figure 2. Long description

The Sankey diagram illustrates the relationships between various models, objective functions, and constraints used to operationalize acceptability in food systems. The models include linear, non-linear, combination, multi-objective, non-linear multi-objective, quadratic, data envelope analysis, hierarchical model with linear functions, and unclear models. The objective functions are categorized into minimizing deviation from observed diet, constraining on individual foods or food groups, and other functions such as nutritional score, environmental impact, and cost. The constraints include boundaries, limiting deviation, repetition of dishes or ingredients, substitution foods grouped together, serving size, solid-liquid ratio, none, and weighting. The diagram visually represents how these elements interconnect to achieve sustainable and acceptable food systems.

Navigate back to Figure 2..

## Table 6. Long description

A table summarizing the impacts of modeling for acceptability in dietary optimization studies across eighteen studies. The table has 18 rows and 8 columns. The columns are labeled as Study, Country, Model section, Objective function, Constraints, Acceptability measurement, impact on diet, and Environmental impact. The rows detail various studies, their respective countries, model sections, objective functions, constraints, acceptability measurements, impacts on diet, and environmental impacts. Diets modeled for acceptability generally resulted in more plant-based compositions with smaller amounts of animal-based products, particularly chicken, fish, and eggs, and a complete to partial reduction in ruminant meats such as beef, compared to observed diets of the study population. Incorporating acceptability into diets did not drastically compromise the nutritional content of the modeled output and in the studies that measured acceptability resulted in increased overall health or nutritional score compared to observed diets, but not always compared to other models with stricter nutritional or environmental constraints.

Navigate back to Table 6..
