# Supplementary material for: Application of acceptability in dietary optimisation models: a systematic review
Source: Public Health Nutr. 2026 May 22;29(1):e147. doi: 10.1017/S1368980026102717 (PMC13434501; doi:10.1017/S1368980026102717)
Supplement: Supplementary file 1 [file S1368980026102717sup001.docx]

# **Supplementary materials: Application of acceptability in dietary optimisation models – a systematic review.**

**Table S1.** Search terms and hits recorded for search in April 2023

| **#** | **Query** | **Results from 23 May 2023** |
| --- | --- | --- |
| 1 | (diet or diets or dietary).mp. [mp=ti, ab, hw, tn, ot, dm, mf, dv, kf, fx, dq, bt, nm, ox, px, rx, an, ui, sy, ux, mx] | 1,412,235 |
| 2 | (modelling or modeling).mp. [mp=ti, ab, hw, tn, ot, dm, mf, dv, kf, fx, dq, bt, nm, ox, px, rx, an, ui, sy, ux, mx] | 827,636 |
| 3 | (optimisation or optimization).mp. [mp=ti, ab, hw, tn, ot, dm, mf, dv, kf, fx, dq, bt, nm, ox, px, rx, an, ui, sy, ux, mx] | 430,500 |
| 4 | (optimise or optimize).mp. [mp=ti, ab, hw, tn, ot, dm, mf, dv, kf, fx, dq, bt, nm, ox, px, rx, an, ui, sy, ux, mx] | 300,213 |
| 5 | 2 or 3 or 4 | 1,480,466 |
| 6 | linear programming.mp. [mp=ti, ab, hw, tn, ot, dm, mf, dv, kf, fx, dq, bt, nm, ox, px, rx, an, ui, sy, ux, mx] | 3,526 |
| 7 | 5 or 6 | 1,482,232 |
| 8 | 1 and 7 | 28,611 |
| 9 | acceptab*.mp. [mp=ti, ab, hw, tn, ot, dm, mf, dv, kf, fx, dq, bt, nm, ox, px, rx, an, ui, sy, ux, mx] | 452,234 |
| 10 | 8 and 9 | 808 |
| 11 | limit 10 to yr="2013 -Current" | 613 |
| 12 | limit 11 to human | 519 |
| 13 | limit 12 to English language | 519 |
| 14 | remove duplicates from 13 | 340 |

**Table S2.** Search terms and hits recorded for search in May 2024

| **#** | **Query** | **Results from 11 April 2024** |
| --- | --- | --- |
| 1 | (diet or diets or dietary).mp. [mp=ti, ab, hw, tn, ot, dm, mf, dv, kf, fx, dq, bt, nm, ox, px, rx, an, ui, sy, ux, mx] | 1,481,957 |
| 2 | (modelling or modeling).mp. [mp=ti, ab, hw, tn, ot, dm, mf, dv, kf, fx, dq, bt, nm, ox, px, rx, an, ui, sy, ux, mx] | 873,647 |
| 3 | (optimisation or optimization).mp. [mp=ti, ab, hw, tn, ot, dm, mf, dv, kf, fx, dq, bt, nm, ox, px, rx, an, ui, sy, ux, mx] | 461,916 |
| 4 | (optimise or optimize).mp. [mp=ti, ab, hw, tn, ot, dm, mf, dv, kf, fx, dq, bt, nm, ox, px, rx, an, ui, sy, ux, mx] | 323,925 |
| 5 | 2 or 3 or 4 | 1,575,397 |
| 6 | linear programming.mp. [mp=ti, ab, hw, tn, ot, dm, mf, dv, kf, fx, dq, bt, nm, ox, px, rx, an, ui, sy, ux, mx] | 3,690 |
| 7 | 5 or 6 | 1,577,227 |
| 8 | 1 and 7 | 30,933 |
| 9 | acceptab*.mp. [mp=ti, ab, hw, tn, ot, dm, mf, dv, kf, fx, dq, bt, nm, ox, px, rx, an, ui, sy, ux, mx] | 477,259 |
| 10 | 8 and 9 | 897 |
| 11 | limit 10 to yr="2023 - 2024" | 130 |
| 12 | limit 11 to human | 109 |
| 13 | limit 12 to English language | 109 |
| 14 | remove duplicates from 13 | 71 |

**Table S3.** Search terms and hits recorded for search in June 2025

| **#** | **Query** | **Results from 11 June 2025** |
| --- | --- | --- |
| 1 | (diet or diets or dietary).mp. [mp=ti, ab, hw, tn, ot, dm, mf, dv, kf, fx, dq, bt, nm, ox, px, rx, an, ui, sy, ux, mx] | 1,589,849 |
| 2 | (modelling or modeling).mp. [mp=ti, ab, hw, tn, ot, dm, mf, dv, kf, fx, dq, bt, nm, ox, px, rx, an, ui, sy, ux, mx] | 954,160 |
| 3 | (optimisation or optimization).mp. [mp=ti, ab, hw, tn, ot, dm, mf, dv, kf, fx, dq, bt, nm, ox, px, rx, an, ui, sy, ux, mx] | 517,004 |
| 4 | (optimise or optimize).mp. [mp=ti, ab, hw, tn, ot, dm, mf, dv, kf, fx, dq, bt, nm, ox, px, rx, an, ui, sy, ux, mx] | 373,106 |
| 5 | 2 or 3 or 4 | 1,748,666 |
| 6 | linear programming.mp. [mp=ti, ab, hw, tn, ot, dm, mf, dv, kf, fx, dq, bt, nm, ox, px, rx, an, ui, sy, ux, mx] | 3,970 |
| 7 | 5 or 6 | 1,750,621 |
| 8 | 1 and 7 | 35,114 |
| 9 | acceptab*.mp. [mp=ti, ab, hw, tn, ot, dm, mf, dv, kf, fx, dq, bt, nm, ox, px, rx, an, ui, sy, ux, mx] | 516,929 |
| 10 | 8 and 9 | 1008 |
| 11 | limit 10 to yr="2023 - 2024" | 141 |
| 12 | limit 11 to human | 128 |
| 13 | limit 12 to English language | 128 |
| 14 | remove duplicates from 13 | 87 |

**Table S4.** Definitions of three paths utilised for data extraction on defining acceptability. Adapted from Sobal and Bisogni (31).

| Path | Definition |
| --- | --- |
| Translation | The use and/or adaptation of existing perspective. |
| Deduction | The process whereby experts use their own expertise, experiences, and observations to develop new models or definitions. |
| Induction | The use of information from citizens to ground models or frameworks in the perspective of food citizens. |

**Table S5. Six-step framework for thematic analysis, adapted from Braun & Clark (2006)**

| Steps | Description of the authors’ methodological approach |
| --- | --- |
| Step 1: Become familiar with data | All studies read by C.B. minimum three times and quantitative and qualitative data extracted, 10% of studies read by M.A.M independently and any issues discussed with B.dR. |
| Step 2: Generate codes | C.B. coding features of qualitative data across entire dataset. |
| Step 3: Search for themes | C.B., gathered all data relevant to each theme in excel document. |
| Step 4: Reviewing themes | C.B., M.A.M and B.dR reviewed and discussed generated themes. See Table S5 for Evolutionary process of thematic analysis |
| Step 5: Defining themes | C.B. refined themes and names following discussions. |
| Step 6: Writing-up | C.B. identified and wrote about relevant themes for results and discussions. All authors critically reviewed, revised and approved the manuscript. |

**Table S6.** Evolutionary process of thematic analysis

| Extracts from the data | Potential themes and meaningful groupings | Final themes and sub-themes | Definition or Application of Acceptability |
| --- | --- | --- | --- |
| "Cultural acceptability has to do with local foods and local agricultural history." (pg. 697)(2)  "…sociocultural acceptability" (pg. 877)(3) | Local food, history, social | **Cultural** | Definition |
| "…acceptability of the diets (via. "more familiar meals")…" (pg. 6)(5) | Familiarity, already known, recognised, common | **Familiar** | Definition |
| "To avoid unrealistic dietary changes…" (pg. 953)(6) | Realistic, contextual foods, accessible, realistic vs unrealistic | **Feasible** | Definition |
| "The theoretical acceptability of the modelled diets was maintained by constraints limiting:…" (pg. 179)(13)  "Food acceptability referred to the maximum theoretically acceptable changes in food quantities, compared with the current observed diet." (pg. 3)(14) | Theoretical application of acceptability with constraints | **Theoretical** | Definition |
| "…acceptable in a variety of contexts." (pg. 10)(10)  "...cultural habits are satisfied by considering the country-specific structure of the meals and an appropriate set of dishes to compose the menu.” (pg. 6)(11)  “After all, different regions have different definitions of food culture and different quantitative methods, so it is necessary to make sustainable dietary recommendations according to the regional and cultural environments…” (pg. 9)(12) | Country specific meals, acceptability can change based on context | **Context specific** | Definition |
| "Fourth, the acceptability of some recommendations might be low for different reasons (palatability, convenience, habits, medical conditions, etc.) (4547), because few women reported consuming” (pg. 1588) (15) | Palatability part of acceptability | **Palatable** | Definition |
| "…it also increases cultural acceptability by staying as close as possible to each observed diet and accounts for personal nutritional requirements." (pg. 2) (Rocabois et al 2022)  "cultural acceptability (i.e., the diet was designed to be as close as possible to the existing individual's diet)…" (pg. 2)(4) | Existing diet, minimise distance, close as possible | **Data inputs**  Sub-theme  **Closest to the existing diet** | Application |
| "…high amounts of 463 vegetables, fruits, wholegrain products and legumes may be an acceptable trade-off for the general 464 population, as it is close to the dietary guidelines…" (pg. 19)(8) | Dietary guidelines acceptable for the general population | **Data inputs**  Sub-theme  **Closest to dietary guidelines** | Application |
| but this remains simplistic at a time where eating habits change very quickly." (pg. 179)(16)  "Cultural acceptability has to do with local foods and local agricultural history." (pg. 697)(2)  "However, this might not be acceptable or feasible in South India, where rice is the major staple food, in contrast with the North, where wheat is the major staple." (pg. 7701)(17) | Changing eating habits, food habits related to local culture or history, | **Data inputs**  Sub-theme  **Context specific dietary sources** | Application |
| “The metric for changes is operationalized by a penalty score which, in turn, reflects the popularity of each food product.” (pg. 703)(18)  “…but methodological the programming retains as many products as possible and if changes needed, recently popular, frequently consumed, products are favored…” (688)(2) | Popular food items are acceptable | **Data inputs**  Sub-theme  **Popular or familiar foods** | Application |
| "We distinguished three types of item: the initially consumed items, the non-consumed items that can be added to the diet and those that cannot be added to the diet for health or cultural reasons (as meat or sweet food). The first two types of item were included in the optimization model; thus, the number of items in the model depended on each participant." (pg. 180)(16) | Cultural foods | **Data inputs**  Sub-theme  **Cultural or religiously relevant foods** | Application |
| “…and for some countries, abandoning the currently consumed foods in favor of other foods is influenced not only by the dietary habits, but also by traditional culture and food availability." (pg. 6)(19) | Acceptability related to availability of foods | **Data inputs**  Sub-theme  **Available foods** | Application |
| "In particular… we adopted a conservative approach based on the hypotheses that individuals, especially those with low income, were more likely to maintain familiar dietary patterns within their habitual caloric intake." (pg. 15)(20)  "…it also increases cultural acceptability by staying as close as possible to each observed diet and accounts for personal nutritional requirements." (pg. 2) (Rocabois et al 2022) | Income groups, demographics, nutritional requirements | **Data inputs**  Sub-theme  **Demographic information** | Application |
| "..we assumed that large shifts in diets will be needed, probably beyond what currently would be considered culturally acceptable." (pg. 291)(21)  "…the used method is that is addresses acceptability in a very natural way as opposed to LP that…" (pg. 2)(11) | Large changes not acceptable, constraints, model is better for acceptability | **Model elements**  Sub-theme  **The quantity and quality of change** | Application |
| "Taking an individual approach to dietary change…illustrates the complexity and many different ways diets can be optimised..." (pg. 8)(22)  "To consider the food preference (cultural acceptability) of the studied population…" (pg. 1954)(23) | Individual approach, specific population, audience affects approach | **Model elements**  Sub-theme  **The audience** | Application |
| “…This term refers to boundaries in which the optimized diet can deviate from the observed mean intake. These limitations help avoid a diet that is culturally or socially unacceptable.” (pg. 1540)(24)  "…acceptability constraints aim to minimize the deviation from current dietary behavior." (pg. 532)(25) | Boundaries to avoid or limit changes | **Model elements**  Sub-theme  **Boundaries** | Application |
| "… incorporating the available evidence on substitution between different food groups…) (pg. 255)(26) | Food group substitution | **Model elements**  Sub-theme  **Substitutability** | Application |
| “… equal weighting was given to each food, which might not be considered as acceptable by the consumer.” (pg. 16)(27)  "Food acceptability referred to the maximum theoretically acceptable changes in food quantities, compared with the current observed diet." (pg. 3)(14) | Weighting of individual foods, | **Model elements**  Sub-theme  **Quantity** | Application |
| "We have shown that more stringent GHGE targets and more stringent eating patterns reduce the diversity of possible diets, potentially limiting their acceptability" (pg. 1345)(28)  “…but this diet was unrealistic due to limited food diversity.” (pg.289)(29)  “To obtain a varied menu, recipes corresponding to first and second courses cannot be served more than once in the entire menu.” (3)(11) | Diversity of diet limit acceptability, diversity of food, diversity of choice | **Model elements**  Sub-theme  **Diversity and variety** | Application |
| “… we mainly consider the food consumption elasticity of CP, pork, beef and mutton, instead of all food categories, because consumption elasticity of these items is b5% (see Table A.5 for the method and results), meaning that residents are unwilling to change their consumption habits for these foods.” (pg. 4)(12)  “…(using the expenditure share and price elasticity of each food group as a proxy for how acceptable changes to the consumption of each food would be to the UK population).” (262)(26) | Consumption elasticity, price elasticity, consumption changes | **Model elements**  Sub-theme  **Elasticity** | Application |
| "The latter option might be more environmentally efficient and also leave more room to keep the diets closer to current patterns and thus more culturally acceptable." (pg. 7700)(17) | Current dietary patterns | **Model elements**  Sub-theme  **Meal or dietary patterns** | Application |
| “Acceptability is achieved by limiting in the menu the repetitions of each recipe or those of recipes in the same food group.” (pg. 6)(11)  “…linear programming can facilitate the definition of nutritionally healthy, environmentally friendly, and culturally acceptable diets, using the Low Lands as an illustrative example.” (pg. 688)(2) | Dietary pattern, developing menus | **Output**  Sub-theme  **Output type** | Application |
| “This partial shift is in line with the food choices of a growing number of consumers identifying themselves as meat reducers or flexitarians(39), which confirms the feasibility of adopting our proposed diets.” (pg. 572)(30)  “The results of our questionnaire also confirmed the residents' unique preferences of meat consumption. According to the preference score for different meats (including seafood), the mutton has the highest score of 6.76 (out of 10 points, the higher the score, the more preference), and 74% of the respondents rated it higher than 5 points.” (pg. 8)(12) | Diet in line with current food choices or trends; using survey results to identify willingness to change | **Output**  Sub-theme  **Evaluation** | Application |

**References**

1.	Braun V, Clarke V. Using thematic analysis in psychology. Qual Res Psychol. 2006 Jan 1;3(2):77–101.

2.	van Dooren C, Aiking H. Defining a nutritionally healthy, environmentally friendly, and culturally acceptable Low Lands Diet. Int J Life Cycle Assess. 2016 May 1;21(5):688–700.

3.	Lucas E, Guo M, Guillén-Gosálbez G. Optimising diets to reach absolute planetary environmental sustainability through consumers. Sustain Prod Consum. 2021 Oct 1;28:877–92.

4.	Gazan R, Vieux F, Lluch A, de Vriese S, Trotin B, Darmon N. Individual Diet Optimization in French Adults Shows That Plant-Based “Dairy-like” Products May Complement Dairy in Sustainable Diets. Sustainability. 2022 Jan;14(5):2817.

5.	Wilson N, Nghiem N, Ni Mhurchu C, Eyles H, Baker MG, Blakely T. Foods and Dietary Patterns That Are Healthy, Low-Cost, and Environmentally Sustainable: A Case Study of Optimization Modeling for New Zealand. PLoS ONE. 2013 Mar 27;8(3):e59648.

6.	Vieux F, Perignon M, Gazan R, Darmon N. Dietary changes needed to improve diet sustainability: are they similar across Europe? Eur J Clin Nutr. 2018;72(7):951–60.

7.	Kanellopoulos A, Gerdessen JC, Ivancic A, Geleijnse JM, Bloemhof-Ruwaard JM, Veer P van’t. Designing healthier and acceptable diets using data envelopment analysis. Public Health Nutr. 2020 Sep;23(13):2290–302.

8.	Kesse-Guyot E, Allès B, Brunin J, Fouillet H, Dussiot A, Mariotti F, et al. Nutritionally adequate and environmentally respectful diets are possible for different diet groups: an optimized study from the NutriNet-Santé cohort. Am J Clin Nutr. 2022 Dec 19;116(6):1621–33.

9.	Sobhani SR, Edalati S, Eini-Zinab H, Kennedy G, Omidvar N. A comparative analysis of sustainability of the usual food intakes of the Iranian population, Iranian food-based dietary guidelines, and optimized dietary models. Front Sustain Food Syst [Internet]. 2022 [cited 2023 Jun 27];6. Available from: https://www.frontiersin.org/articles/10.3389/fsufs.2022.838741

10.	Thompson S, Gower R, Darmon N, Vieux F, Maillot M, Murphy-Bokern D. Livewell for life. A balance of healthy and sustainable food choices for France, Spain, and Sweden [Internet]. 2013 [cited 2023 Jun 21]. Available from: https://hal.science/hal-01601768

11.	Benvenuti L, De Santis A, Cacchione P. Multi-indicator design and assessment of sustainable diet plans. J Clean Prod. 2021 Sep 1;313:127699.

12.	Yin J, Yang D, Zhang X, Zhang Y, Cai T, Hao Y, et al. Diet shift: Considering environment, health and food culture. Sci Total Environ. 2020 Jun 1;719:137484.

13.	Maillot M, Darmon N. Testing the nutritional relevance of food-based dietary guidelines with mathematical optimisation of individual diets. Nutr Bull. 2020;45(2):175–88.

14.	Verly-Jr E, Sichieri R, Darmon N, Maillot M, Sarti FM. Planning dietary improvements without additional costs for low-income individuals in Brazil: linear programming optimization as a tool for public policy in nutrition and health. Nutr J. 2019 Jul 20;18:40.

15.	Brouzes CMC, Darcel N, Tomé D, Bourdet-Sicard R, Youssef Shaaban S, Gamal El Gendy Y, et al. Local Foods Can Increase Adequacy of Nutrients Other than Iron in Young Urban Egyptian Women: Results from Diet Modeling Analyses. J Nutr. 2021 Jun 1;151(6):1581–90.

16.	Seconda L. Conservative to disruptive diets for optimizing nutrition, environmental impacts and cost in French adults from the NutriNet-Santé cohort. 2021;

17.	Chaudhary A, Krishna V. Country-Specific Sustainable Diets Using Optimization Algorithm. Environ Sci Technol. 2019 Jul 2;53(13):7694–703.

18.	Tyszler M, Kramer G, Blonk H. Just eating healthier is not enough: studying the environmental impact of different diet scenarios for Dutch women (31–50 years old) by linear programming. Int J Life Cycle Assess. 2016 May 1;21(5):701–9.

19.	Liu X, Xin L, Li X. The global nutrition can be greatly improved with diet optimization. Resour Conserv Recycl. 2024 Mar 1;202:107343.

20.	Maillot M, Vieux F, Delaere F, Lluch A, Darmon N. Dietary changes needed to reach nutritional adequacy without increasing diet cost according to income: An analysis among French adults. PLOS ONE. 2017 Mar 30;12(3):e0174679.

21.	Mazac R. Incorporation of novel foods in European diets can reduce global warming potential, water use and land use by over 80%.

22.	Horgan GW, Perrin A, Whybrow S, Macdiarmid JI. Achieving dietary recommendations and reducing greenhouse gas emissions: modelling diets to minimise the change from current intakes. Int J Behav Nutr Phys Act. 2016 Apr 7;13(1):46.

23.	Eini-Zinab H, Sobhani SR, Rezazadeh A. Designing a healthy, low-cost and environmentally sustainable food basket: an optimisation study. Public Health Nutr. 2021 May;24(7):1952–61.

24.	Santos Q dos, Sichieri R, Darmon N, Maillot M, Verly-Junior E. Food choices to meet nutrient recommendations for the adult Brazilian population based on the linear programming approach. Public Health Nutr. 2018 Jun;21(8):1538–45.

25.	Chaudhary A, Krishna V. Region-specific nutritious, environmentally friendly, and affordable diets in India. One Earth. 2021 Apr 23;4(4):531–44.

26.	Green R, Milner J, Dangour AD, Haines A, Chalabi Z, Markandya A, et al. The potential to reduce greenhouse gas emissions in the UK through healthy and realistic dietary change. Clim Change. 2015 Mar 1;129(1):253–65.

27.	Barré T, Perignon M, Gazan R, Vieux F, Micard V, Amiot MJ, et al. Integrating nutrient bioavailability and co-production links when identifying sustainable diets: How low should we reduce meat consumption? PLOS ONE. 2018 Feb 14;13(2):e0191767.

28.	Broekema R, Tyszler M, van ’t Veer P, Kok FJ, Martin A, Lluch A, et al. Future-proof and sustainable healthy diets based on current eating patterns in the Netherlands. Am J Clin Nutr. 2020 Nov 1;112(5):1338–47.

29.	Song G, Li M, Fullana-i-Palmer P, Williamson D, Wang Y. Dietary changes to mitigate climate change and benefit public health in China. Sci Total Environ. 2017 Jan 15;577:289–98.

30.	Mertens E, Kuijsten A, Kanellopoulos A, Dofková M, Mistura L, D’Addezio L, et al. Improving health and carbon footprints of European diets using a benchmarking approach. Public Health Nutr. 2021 Feb;24(3):565–75.

31.	Sobal J, Bisogni CA. Constructing Food Choice Decisions. Ann Behav Med. 2009 Dec 1;38(suppl_1):s37–46.

Count

| Dietary data sources | Count |
| --- | --- |
| National surveys | 46 |
| Local surveys | 16 |
| Combination | 3 |
| National statistics | 2 |
| Food balance sheet | 2 |
| Other sources | 2 |
| Cohort study | 2 |

**Figure S1**. Dietary data sources used by studies in this systematic review.

| Dietary data methodology | Count |
| --- | --- |
| 24-hr recall | 16 |
| Combination | 11 |
| 7-day food diary | 10 |
| Baseline menu | 9 |
| Not clear | 6 |
| Food Balance Sheet | 5 |
| 4-day food diary | 4 |
| 2-day food diary | 3 |
| 3-day food diary | 3 |
| Food Frequency Questionnaire | 2 |
| National Statistics | 2 |
| 30-day food diary | 1 |
| Supermarket supply | 1 |

**Figure S2**. Dietary data methodologies used by studies in this systematic review.

| Year intervals | Count |
| --- | --- |
| 0-3 years | 13 |
| 4-7 years | 26 |
| 8-11 years | 17 |
| 12 - 15 years | 9 |
| 16+ years | 1 |
| Not clear | 7 |

**Figure S3.** Different in years between year of dietary data collection and publication year.

**Figure S4.** Map of countries represented by studies included in systematic review.

**Figure S5.** Type of optimisation models used by studies in the systematic review.

| Minimising deviation objective functions | Count |
| --- | --- |
| Minimise deviation | 17 |
| Minimise absolute deviation | 5 |
| Minimise relative deviation | 4 |
| Minimise sum of squares | 5 |
| Minimising sum of absolute deviation between relative weight | 1 |

**Figure S6.** Type of minimisation deviation objective functions.

| Types of objective function | Count |
| --- | --- |
| Minimise deviation | 31 |
| No acceptability objective function | 17 |
| Combination | 2 |

**Figure S7.** Categorisation of objective functions used by studies in systematic review.

**Figure S8.** Number of studies using the three pathways (translation, deduction, and induction) to operationalise different components of dietary optimisation.

**References**

30.	Sobal J, Bisogni CA. Constructing Food Choice Decisions. *Annals of Behavioral Medicine*. 2009;38(suppl_1):s37-s46. doi:10.1007/s12160-009-9124-5.
